# Microfluidic Systems for Blood and Blood Cell Characterization

**DOI:** 10.3390/bios13010013

**Published:** 2022-12-22

**Authors:** Hojin Kim, Alexander Zhbanov, Sung Yang

**Affiliations:** 1 Department of Mechatronics Engineering, Dongseo University, Busan 47011, Republic of Korea; 2 School of Mechanical Engineering, Gwangju Institute of Science and Technology (GIST), Gwangju 61005, Republic of Korea; 3 Department of Biomedical Science and Engineering, Gwangju Institute of Science and Technology (GIST), Gwangju 61005, Republic of Korea

**Keywords:** microfluidic systems, blood, blood cells, physical properties, biophysical characteristics

## Abstract

A laboratory blood test is vital for assessing a patient’s health and disease status. Advances in microfluidic technology have opened the door for on-chip blood analysis. Currently, microfluidic devices can reproduce myriad routine laboratory blood tests. Considerable progress has been made in microfluidic cytometry, blood cell separation, and characterization. Along with the usual clinical parameters, microfluidics makes it possible to determine the physical properties of blood and blood cells. We review recent advances in microfluidic systems for measuring the physical properties and biophysical characteristics of blood and blood cells. Added emphasis is placed on multifunctional platforms that combine several microfluidic technologies for effective cell characterization. The combination of hydrodynamic, optical, electromagnetic, and/or acoustic methods in a microfluidic device facilitates the precise determination of various physical properties of blood and blood cells. We analyzed the physical quantities that are measured by microfluidic devices and the parameters that are determined through these measurements. We discuss unexplored problems and present our perspectives on the long-term challenges and trends associated with the application of microfluidics in clinical laboratories. We expect the characterization of the physical properties of blood and blood cells in a microfluidic environment to be considered a standard blood test in the future.

## 1. Introduction

Blood circulates throughout the body, containing extensive information about the body’s state of health and disease, and laboratory blood tests remain the most common diagnostic procedure. Test results should help doctors diagnose a disease and evaluate a patient’s response to treatment. It is generally accepted that 70% of diagnoses are made based on blood tests [1]; conventional clinical laboratories routinely perform hundreds of them with different blood test tubes being sent to hematology, biochemistry, and microbiology departments [2].

Microfluidic technology facilitates the development of modern tools that use significantly lower blood volumes and provide shorter analysis times than conventional laboratory methods. Added benefits include the small volumes of reagent required and less biological and chemical waste. Miniature microfluidic chips with advanced biosensors and computing systems offer promising prospects for use in point-of-care (POC) diagnostics and personalized medicine [3,4], a field of microfluidics that has been growing exponentially. The number of reviews on this topic alone exceeds hundreds of publications per year.

This review is focused on the latest advances in microfluidic systems for obtaining the physical properties and biophysical characteristics of blood and blood cells. Added emphasis is placed on multifunctional platforms that combine several such microfluidic technologies.

Common blood tests include just a few parameters that assess blood and blood cell physical properties. One of these tests is the full blood count (FBC) test, which includes red blood cell (RBC) count, white blood cell (WBC) count, platelet (PLT) count, mean corpuscular volume (MCV), mean corpuscular hemoglobin (MCH) count, mean corpuscular hemoglobin concentration (MCHC), hematocrit or volume percentage of RBCs in whole blood (HCT), amongst other determinations.

In modern laboratories, Coulter automated hematology analyzers that utilize light scattering and electrical impedance are typically used for FBC tests. These automatic hematology analyzers have now been reproduced in microfluidics [5,6,7,8]. When reviewing microfluidic flow cytometry, Béné reported that it would likely revolutionize the profession of hematology biologists [9]. Recent advances in microfluidic flow cytometry have been reviewed by Yang et al. [10], Daguerre et al. [11], Kuan and Huang [12], Honrado et al. [13], and Zhu et al. [14].

Plasma viscosity is another physical property that refers to the inflammatory panel of biochemical analysis. We classified the erythrocyte sedimentation rate (ESR) from the same inflammatory panel as a biophysical characteristic but not as a physical property of blood. The ESR is the average speed of the plasma–blood interface movement in a vertical test tube of a certain height and diameter over 1 h. Consequently, the ESR reflects both the properties of the blood and the properties of the measuring device. Similarly, we classified the deformation index (DI), deformability, the aggregation index (AI), the aggregability of blood cells, blood impedance, and other parameters dependent on specific devices and conditions as biophysical characteristics.

One of the objectives of this review is to determine the physical quantity that is measured by a particular device—for example, transit time, light intensity, and velocity profile—and the parameter that is determined by it—for example, DI, AI, and viscosity. 

**Figure 1:**
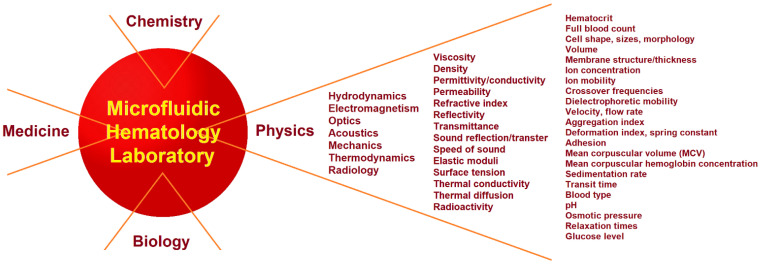
Contribution of physics to a microfluidic hematology laboratory.

The branches of physics related to the measurement of the physical properties and biophysical characteristics of blood are outlined in Figure 1.

Radiometric detection methods for blood characterization will not be discussed in this review, as Liu and Lan have already reviewed microfluidic radiobioassays [15]. Additionally, genotype characterization and analysis of gene expression patterns in circulating tumor cells (CTCs) are outside the scope of this review. For a comprehensive description of CTC analysis in terms of detecting genetic mutations and gene expressions, please refer to the work of Jackson et al. [16] and Yu et al. [17].

The structure of this review is as follows: the role of physics in a microfluidic hematology laboratory is discussed in Section 2, the various forces acting on particles in microfluidic channels are described in Section 3, combinations of different techniques in a single platform for blood cell separation are reviewed in Section 4, microfluidic cytometry and biophysical characteristics of blood are discussed in Section 5, and measurement of the physical properties of blood and blood cells are discussed in Section 6. Finally, in Section 7, we provide an overview on the future development of a hematology laboratory on a chip. A list of abbreviations used is placed at the end of the review.

## 2. Physics and Physical Experiments in Hematology

### 2.1. Clinical Hematology Laboratory

Routine blood tests are carried out in the departments of hematology, biochemistry, and microbiology.

Hematological examinations include complete blood counts (erythrocyte, leukocyte, and thrombocyte counts) and the determination of hemoglobin levels and hematocrit ratios, etc.

Biochemical analysis of blood identifies the biological and chemical compounds produced by organs and cellular processes. Such tests consist of several panels, including basic metabolic (or electrolyte) panels (electrolytes, calcium, glucose, sodium, potassium, carbon dioxide, chloride, blood urea nitrogen, and creatinine), complete (or comprehensive) metabolic panels (albumin, total protein, alkaline phosphatase, alanine aminotransferase, aspartate aminotransferase, and bilirubin), lipid panels (high-density lipoproteins and low-density lipoproteins), inflammatory panels (plasma viscosity, erythrocyte sedimentation rate, and C-reactive proteins), amongst others.

Microbiology laboratory tests check blood samples to identify viruses, bacteria, yeasts, fungi, parasites, and other microorganisms. Tests for bacteria and viruses have different testing principles. For bacteriological examinations, colonies of a wide variety of microorganisms obtained from biomaterials—such as blood—are grown on a universal nutrient media. Such microfluidic platforms for cell cultures have been recently reviewed by Coluccio et al. [18]. This is not the case for viruses, which are not cellular life forms and can only reproduce in living cells using cellular structures for protein synthesis and viral genetic material. Clinical methods for recognizing viruses include enzyme-linked immunosorbent assays, fluorescence in situ hybridization, polymerase chain reaction, etc. Darwish et al. [19] reviewed the application of microfluidic tools for the diagnosis of the dengue virus. Qureshi and Niazi reviewed the application of microfluidic platforms for both bacterial and viral infections [20].

### 2.2. Physical Measurements in a Microfluidic Hematology Laboratory

More than three decades of research have resulted in the development of various microfluidic devices for rapid and accurate clinical diagnosis, where the role of physics is increasing markedly. With its compelling clinical advantages, liquid biopsy has emerged as a novel method for diagnosing, monitoring, and treating cancer using precision medicine [21,22,23,24]. Belotti and Lim reviewed recent advances in microfluidics for the fluid biopsy of not only the blood from patients with cancer, but also other body fluids, such as urine, saliva, pleural effusion, and cerebrospinal fluid [25]. Cheng et al. [26] summarized the progress in microfluidic analysis of extracellular vesicles secreted by cells into the bloodstream and other body fluids for cancer diagnosis. Cho et al. [27] presented the latest advances in microfluidic chips for cancer diagnosis and recurrence prediction by detecting CTCs and circulating cancer stem cells. As the potential of microfluidics is evident, microfluidic devices are now being commercialized successfully. Commercially available platforms for the detection of CTCs have been reviewed by Chen et al. [28] and Habli et al. [29]. Further, Sachdeva et al. [30] analyzed the commercial landscape of microfluidic POC testing.

Currently, microfluidic devices can reproduce a large volume of laboratory tests, and the designs for each test are being improved continuously. Presently, a growing trend towards combining multiple tests on a single microfluidic chip is being seen, which has been highlighted as a promising research avenue in several recent reviews [31,32,33,34,35]. As an early manifestation of this trend, van Berkel et al. [36] demonstrated a generic microfluidic platform for rapid POC blood cell analysis (2011). The functionality of this platform included lysis and the quenching of blood for WBC counts, dilution of blood for RBC/PLT counts, and impedance cytometry.

Certain steps have also been taken to combine hematology, biochemistry, and microbiology tests on a single chip. Microfluidics provides an opportunity not only for hematological, biological, and microbiological analyses, but also for the measurement of the many other physical properties of blood and blood cells. A list of these physical properties includes the electrical conductivity and permittivity of the cell membrane and interior, membrane elasticity, surface tension, and refractive indices, among others. The contribution of physics to a microfluidic hematology laboratory is shown in Figure 1. The physical properties of blood and blood cells can be obtained by combining hydrodynamic, optical, electromagnetic, and/or acoustic methods into a single microfluidic channel. The boundary between physical, chemical, and biological properties is arbitrary, with some properties being interdisciplinary. The challenges of developing lab-on-a-chip platforms for analyzing both the physical properties and biophysical characteristics of cancer with single-cell resolution were summarized in a recent review by Shukla et al. [37].

## 3. Theory and Physical Principles

**Figure 2:**
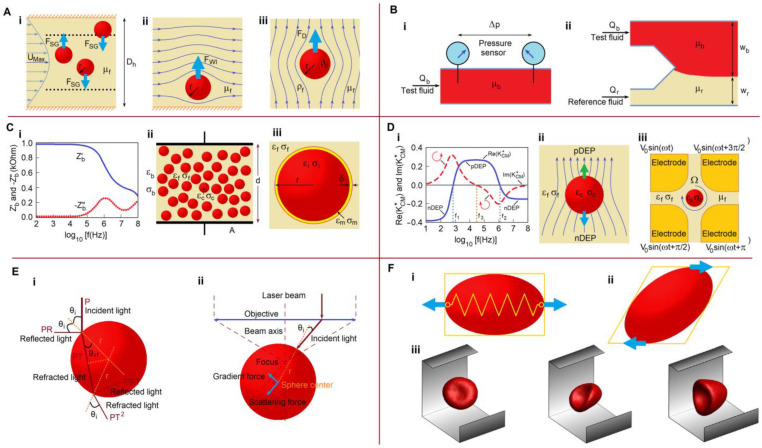
Overview of physical forces acting in microfluidic systems and effective properties of cell materials and cell suspensions. (**A**) Essential forces acting on particles in microfluidic channels: (**i**) shear-gradient lift force, (**ii**) wall-induced lift force, and (**iii**) drag force. (**B**) Direct and indirect methods for measuring viscosity: (**i**) pressure-sensing viscometer and (**ii**) co-flowing stream viscometer. (**C**) Electrochemical impedance spectroscopy of blood: (**i**) real and imaginary parts of the blood impedance, (**ii**) parallel plate measuring cell, and (**iii**) model of the coated spherical particle of an RBC. (**D**) Dielectrophoretic force and electrorotation torque: (**i**) real and imaginary parts of the Clausius–Mossotti factor, (**ii**) positive and negative dielectrophoresis, and (**iii**) four-pole electrode structure for rotating a dielectric particle. (**E**) Optical trapping of spherical particle: (**i**) reflection and refraction of incident light in spherical particle and (**ii**) contribution of an incident ray to scattering and gradient forces. (**F**) Mechanical properties of RBCs: (**i**) Young’s modulus and spring model, (**ii**) shear modulus, and (**iii**) change in the biconcave shape of RBCs at different flow conditions: tumbling, tank-treading, parachute shapes (from left to right). Image (F(**iii**)) was adapted with permission from [41]. Copyright 2019, Biophysical Society.

Determining the physical properties and/or biophysical characteristics of any material requires a measuring system for data acquisition and an appropriate physical theory to convert the obtained data into physical quantities. Biological materials in microfluidic systems can be subjected to hydrodynamic, electromagnetic, acoustic, optical, and mechanical effects or any combination of them. This section aims to describe the various forces and fields acting in microfluidic channels and discuss the properties that can be extracted from experimental data. An incomplete list of the physical properties and biophysical characteristics of blood and blood cells is shown in Figure 1. The primary forces acting on cells in microfluidic systems are shown in Figure 2. For simplicity, all blood cells are considered to be spherical particles, since their shape does not affect the fundamental relationships between the physical properties of cells and the acting forces and fields.

### 3.1. Hydrodynamic Forces in Microfluidic Systems

#### 3.1.1. Reynolds Number

All biological cells and fluids in microfluidic channels are exposed to hydrodynamic forces. The Reynolds number describes the boundaries of the flow regimes when a liquid flows in a microchannel and/or particles move in a liquid. The channel Reynolds number (Re*_C_*) represents the ratio of inertial force to viscous force acting within the fluid [38,39,40]:

(1) ReC=ρfUMaxDhμf,

where *ρ_f_*, *U_Max_*, and *μ_f_* are the fluid density, maximum velocity, and dynamic viscosity, respectively, and *D_h_* is the hydraulic diameter of the channel. In the case of a cylindrical channel, *D_h_* is equal to the channel diameter. For a rectangular channel, *D_h_* is defined as:

(2) $$D_{h}=\frac{2hw}{\left(h+w\right)},$$

where *h* and *w* are the height and width of the channel, respectively. In most practical microfluidic devices, Re*_C_* < 100.

The inertial force increases with increasing particle size relative to the channel diameter [38]. The particle Reynolds number (Re*_P_*) includes this hydrodynamic phenomenon to express the motion of a particle in a channel flow [40]:

(3) ReP=ReC(2rDh)2=4ρfUMaxr2μfDh,

where *r* is the particle radius. If Re*_P_* ≥ 1, the inertia of the fluid is significant at the particle scale. 

The other particle Reynolds number (Re*_S_*) can be calculated for a single particle moving through an unlimited volume of liquid [42]:

(4) ReS=2ρfurμf,

where *u* is the mean velocity of the particle relative to the fluid. If a biological cell settles in a liquid under the action of gravity, then Re*_S_* << 1.

#### 3.1.2. Wall Interaction and Shear-Gradient Lift and Drag Forces

The nature of the forces acting in microfluidic channels has been reviewed by Martel and Toner [38]. The primary forces are shown in Figure 2A. A particle in a parabolic velocity field experiences a shear-gradient lift force (*F_SG_*) [38]:

(5) $$F_{SG}=\frac{C_{SG}\rho_{f}U_{Max}^{2}{\left(2r\right)}^{3}}{D_{h}}.$$

Particles near the microchannel walls are subjected to a wall-induced lift force (*F_Wl_*) [38,43]:

(6) $$F_{Wl}=\frac{C_{Wl}\rho_{f}U_{Max}^{2}{\left(2r\right)}^{6}}{D_{h}^{4}},$$

where *C_SG_* and *C_Wl_* are the lift coefficients for the shear-gradient lift and wall-induced lift forces, respectively. The lift coefficients depend on the position of the particle in the channel, Re*_C_*, and the aspect ratio of the rectangular channel.

A particle moving relative to a liquid experiences a drag force, as described by Stokes’ law:

(7) $$F_{D}=6\pi \mu_{f}ru.$$

Many modifications of Equations (5)–(7) are known for non-spherical particle cases, but their functional dependence on physical properties remains the same. Blood is set in motion by a pulsating heartbeat in living organisms. The features of pulsating flow in microfluidic systems are discussed by Dincau et al. [44].

#### 3.1.3. The Force Balance

The hydrodynamic forces considered above contain the following physical parameters characterizing blood and blood cells: plasma viscosity, cell and plasma density, and effective radius of a blood cell. Determining the physical parameters by measuring the hydrodynamic forces in a microchannel directly can be problematic. However, Newton’s first law states that if the net force acting on an object is zero, the velocity of the object remains constant. This makes the use of the force balance equation to determine physical properties possible.

Let us consider the example of a single particle falling in a viscous stagnant fluid under gravity. If the drag force is balanced by the buoyancy force and the effect of gravity, the resulting settling velocity (or terminal velocity *u_t_*) is given by [42]:

(8) $$u_{t}=\frac{2(\rho_{c}-\rho_{f})r^{2}g}{9\mu_{f}}.$$

where *ρ_c_* is the density of the particle (biological cell) and *g* is the gravitational acceleration. Thus, if the terminal velocity is measured experimentally, we should be able to determine one of the physical parameters using Equation (8), provided that all other parameters are known. As another example, suspended particles move along streamlines to equilibrium positions within a microfluidic channel (e.g., the dotted lines shown in Figure 2A(i). A detailed description of this phenomenon is provided in ref. [39]. The measured equilibrium positions or the observed deviations of the trajectories of different particles allow one to determine the relationships between physical parameters.

Laxmi et al. [45] used the combined result of hemodynamic, hydrodynamic, and geometric effects to separate platelets from erythrocytes. In a microchannel, erythrocytes tend to move towards the center of the flow, while the platelets move laterally towards the microchannel walls due to convection and shear diffusion.

#### 3.1.4. Measurement of Whole Blood and Plasma Viscosities

Viscosity measurement in microfluidics can be divided into direct and indirect methods. Direct methods use only the test liquid, whereas indirect methods use a test and a reference liquid. Both methods are based on force balance.

Pressure-sensing viscometers use the direct method with known, predetermined flow rates (Figure 2B(i)). A balance is established between the pressure and viscosity forces via steady blood flow through a straight microfluidic channel. The flow rate (*Q*) of blood through the rectangular channel can be expressed as [46]:

(9) $$Q=\frac{wh^{3}\Delta p}{12\mu_{b}L}\left[1-\frac{192h}{w\pi^{5}}\sum_{n,odd}^{\infty }\frac{1}{n^{5}}\tanh\left(\frac{n\pi w}{2h}\right)\right],$$

where *µ_b_* is the apparent blood viscosity, *L* is the channel length, and Δ*p* is the pressure drop.

The apparent shear rate (${\dot{\gamma }}_{a}$) of a rectangular channel is a linear function of the flow rate, as follows [47,48]:

(10) $${\dot{\gamma }}_{a}=\frac{6Q}{wh^{2}}.$$

Thus, by measuring the flow rate and pressure drop, determining the dependence of viscosity on the shear rate of non-Newtonian fluids is possible. Jung and Yang proposed a device wherein a syringe pump maintained a constant flow rate and the pressure drop was measured using a liquid metal sensor [49].

Co-flowing stream viscometers use the indirect measurement method. Typically, the test and reference fluids are injected at known flow rates (*Q_b_* and *Q_r_*) into a Y-shaped microchannel (Figure 2B(ii)). The location of the liquid–liquid interface determines the viscosity of the test fluid. In the limiting case of a two-dimensional microchannel (*h* << *w*), the unknown viscosity is:

(11) $$\mu_{b}=\mu_{r}\frac{w_{b}Q_{r}}{w_{r}Q_{b}},$$

where the subscripts *Q_b_* and *Q_r_* denote the test and reference fluid, respectively.

Blood viscosity measurement does not require knowledge regarding any additional physical properties of blood, making this area of research particularly attractive for study. Several reviews have discussed viscosity measurement in microfluidics [50,51,52]. Viscosity measurements can be combined with other technologies on one platform to characterize blood and blood cells.

### 3.2. Electromagnetic Characterization of Blood and Blood Cells

#### 3.2.1. Electrochemical Impedance Spectroscopy

Electrochemical impedance spectroscopy (EIS) can be configured with either two, three, or four electrodes. In its most common form, EIS measures the frequency dependence of blood impedance ($Z_{b}^{*}$) between two electrodes:

(12) $$Z_{b}^{*}=\frac{R_{b}}{1+j\omega R_{b}C_{b}}=\frac{R_{b}}{1+(\omega R_{b}C_{b}{)}^{2}}-j\frac{\omega R_{b}^{2}C_{b}}{1+(\omega R_{b}C_{b}{)}^{2}},$$

where *R_b_* and *C_b_* are the resistance and capacitance of the blood sample in the measuring cell for a given electrode configuration, respectively, $j=\sqrt{-1}$ is the imaginary unit, and *ω* is the angular frequency. 

Resistance and reactance are the real and imaginary parts of the impedance, respectively:

(13) Z′b=Re(Zb*)=Rb1+(ωRbCb)2, Z″b=Im(Zb*)=−ωRb2Cb1+(ωRbCb)2.

The typical frequency dependencies of blood resistance and reactance are shown in Figure 2C(i). Note that although the impedance depends on both the properties of the measuring cell and the blood sample, the frequency corresponding to the maximum reactance depends only on the properties of the blood. Thus, this frequency is a physical parameter of blood, and impedance is a biophysical characteristic based on our terminology.

The capacitance and resistance of blood in an ideal parallel plate measuring cell are:

(14) $$C_{b}=\frac{\varepsilon_{b}\varepsilon_{0}A}{d}\;\mathrm{and}\;R_{b}=\frac{d}{\sigma_{b}A},$$

where *ε_b_* and *σ_b_* are the effective relative permittivity and conductivity of blood, respectively, *ε*_0_ is the dielectric constant, *A* is the area of the plates, and *d* is the separation (Figure 2C(ii)).

#### 3.2.2. Effective Properties of a Two-Phase System

The complex relative permittivity (ε*) of any material can be defined as:

(15) $$\varepsilon^{*}=\varepsilon +\frac{\sigma }{j\omega \varepsilon_{0}}=\varepsilon -j\frac{\sigma }{2\pi f\varepsilon_{0}},$$

where *ε* and *σ* are the relative permittivity and conductivity, respectively, and *f* is the ordinary frequency.

Whole blood is a composite material consisting of a liquid matrix (plasma) and suspended inclusions (RBCs, WBCs, and PLTs), with plasma and RBCs predominantly determining the dielectric properties of whole blood. It is assumed that all the inclusions are spherical RBCs randomly distributed in the plasma (Figure 2C(ii)). The Maxwell–Wagner theory provides a reasonable prediction of the dielectric dispersion in a two-phase system [53]:

(16) $$\varepsilon_{b}^{*}=\varepsilon_{f}^{*}\frac{2(1-\phi )\varepsilon_{f}^{*}+(1+2\phi )\varepsilon_{c}^{*}}{(2+\phi )\varepsilon_{f}^{*}+(1-\phi )\varepsilon_{c}^{*}},$$

where $\varepsilon_{b}^{*}$ is the effective complex permittivity of blood, $\varepsilon_{c}^{*}$ is the equivalent complex permittivity of RBCs, $\varepsilon_{f}^{*}$ is the complex permittivity of plasma, and *φ* is the volume fraction of RBCs (HCT = 100*φ*). This equation provides fairly accurate results for a conductive fluid with insulating inclusions, even for a high-volume fraction of inclusions (*φ* ≤ 0.45) [54,55].

The conventional model of an RBC is cytoplasm covered by a single shell or membrane (Figure 2C(iii)). The equivalent complex permittivity of a single-shelled sphere ($\varepsilon_{c}^{*}$) can be expressed as [56]:

(17) $$\varepsilon_{c}^{*}=\varepsilon_{m}^{*}\frac{2\varepsilon_{m}^{*}+\varepsilon_{i}^{*}-2\eta_{\delta }(\varepsilon_{m}^{*}-\varepsilon_{i}^{*})}{2\varepsilon_{m}^{*}+\varepsilon_{i}^{*}+\eta_{\delta }(\varepsilon_{m}^{*}-\varepsilon_{i}^{*})},$$

where $\varepsilon_{i}^{*}$ and $\varepsilon_{m}^{*}$ are the complex permittivities of the erythrocyte cytoplasm and membrane, respectively (Figure 2C(iii)). The ratio of the cytoplasm volume to the whole erythrocyte volume is:

(18) $$\eta_{\delta }={\left(\frac{r-\delta }{r}\right)}^{3},$$

where *r* is the RBC radius and *δ* is the membrane thickness.

Substituting Equation (17) into Equation (16) yields a three-phase system’s effective complex permittivity. This approach can be extended to multiphase systems [53]. Accordingly, Xu et al. [57] considered the dielectric properties of nucleated RBCs using a double spherical shell model.

Thus, electrochemical impedance spectroscopy makes the determination of not only the relative permittivity and conductivity of the plasma (*ε_f_* and *σ_f_*), the cell interior (*ε_i_* and *σ_i_*), and the cell membrane (*ε_m_* and *σ_m_*) possible, but also the ratio of the membrane thickness to the cell radius (*δ*/*r*) and cell volume fraction (*φ*). However, determining these parameters simultaneously based on the experimental impedance spectrum using a fitting algorithm is nearly impossible. The Maxwell–Wagner theory is valid only if the external electric field remains uniform within a sufficiently large ensemble of particles. Therefore, this theory should be applied with great care if the electric field changes noticeably within a single cell. Similar conclusions can be drawn with respect to other analytical and numerical approaches for analyzing impedance spectra.

#### 3.2.3. Dielectrophoretic Forces

The dielectrophoretic (DEP) force (*F_DEP_*) exerted by a non-uniform electric field on a small dielectric cell can be determined in the dipole approximation using the following expression [58]:

(19) FDEP=2πεfε0r3Re(KCM*)∇E02,

where ∇ is the del vector operator and *E*_0_ is the applied electric field. Re(KCM*) is the real part of the Clausius–Mossotti (CM) factor:

(20) $$K_{CM}^{*}=\frac{\varepsilon_{c}^{*}-\varepsilon_{f}^{*}}{\varepsilon_{c}^{*}+2\varepsilon_{f}^{*}}.$$

The equivalent complex permittivity of a single-shelled cell ($\varepsilon_{c}^{*}$) is frequency-dependent according to Equation (17). Consequently, the real part of the CM factor is also frequency-dependent. In some cases, this factor can be negative at low frequencies, becoming positive at mid frequencies, before becoming negative again at high frequencies (Figure 2D(i)).

The real CM factor changes sign for the first time at the first crossover frequency (*f*_1_) and for the second time at the second crossover frequency (*f*_2_) [59,60]. If the real CM factor is positive (Re(KCM*)>0), the cell moves toward the strong field—namely, positive dielectrophoresis (pDEP). Conversely, the cell moves toward the low field if the factor is negative (Re(KCM*)<0)—namely, negative dielectrophoresis (nDEP) (Figure 2D(ii)). From the crossover frequencies—that is, when the cell is not exposed to an electric field—we can obtain the following relationship:

(21) Re(εc*−εf*εc*+2εf*)=0,

which is independent of the electrode configuration and field strength. This phenomenon is particularly useful for characterizing the physical properties of biological cells.

Yildizhan et al. [61] exploited the dielectrophoresis (DEP) force to separate live and dead U937 monocytes. They showed that living U937 monocytes exhibited both nDEP and pDEP behavior in the 50 kHz to 1 MHz range, while dead U937 monocytes were not subjected to DEP forces. The first crossover frequency for the live monocytes was at 150 kHz. Consequently, separation experiments were conducted using 20 V_pp_ signals at 300 kHz.

The dielectric model of a double-shelled cell or a double-shelled cell with vesicle inclusion exhibits more complex real CM factor behavior than the single-shelled sphere shown in Figure 2D(i) [62]. The dielectrophoretic velocity (*v_DEP_*) and the dielectrophoretic mobility (*µ_DEP_*) are related by the following equation [63,64]:

(22) vDEP=−μDEP∇E02=−(ε0εfRe(KCM*)3μf)∇E02.

The membrane relaxation time (*τ_m_*) is given by [64,65]:

(23) $$\tau_{m}=rC_{m}\left(\frac{1}{\sigma_{m}}+\frac{1}{2\sigma_{f}}\right),$$

where *C_m_* is the membrane capacitance of the cell.

Directly measuring the dielectrophoretic force is difficult. Additionally, a similar problem for hydrodynamic forces has already been mentioned. However, the balance between dielectrophoretic and hydrodynamic forces unlocks the possibility of determining the physical properties of cells.

Benhal et al. [66] and Ou et al. [67] recently reviewed the biological applications of DEP at the micro, nano, and molecular scales.

#### 3.2.4. Electrorotation

The four-pole electrode system shown in Figure 2D(iii) generates a rotating electric field, under the action of which a spherical dielectric particle is affected by torque (*T_ROT_*), which can be expressed as [58,68]:

(24) TROT=4πεfε0r3Im(KCM*)E02,

where, Im(KCM*) is the imaginary part of the CM factor. This factor can be positive or negative and can exhibit several crossover frequencies (Figure 2D(i)). The change in sign indicates a change in the direction of rotation. A spherical particle rotating in a viscous fluid with an angular velocity (Ω) is subjected to a torque (*T_D_*) arising from the Stokes drag force:

(25) $$T_{D}=8\pi \mu_{f}\Omega r^{3}.$$

In equilibrium, the angular velocity is constant [68]:

(26) Ω=εfε02μfIm(KCM*)E02.

The frequency dependence of angular velocity provides information on the physical properties of biological cells, whose dielectric characterization using electrokinetics was recently reviewed by Adekanmbi and Srivastava [60]. The balance of the dielectrophoretic and hydrodynamic forces and the positions of cells in microfluidic channels were analyzed in a review paper by Daguerre et al. [11].

#### 3.2.5. Magnetic Force

The magnetic force (*F_MAG_*) affecting a magnetic bead is given by the following [59,69]:

(27) $$F_{MAG}=\chi_{eff}V_{b}\frac{\nabla B^{2}}{2\mu_{0}},$$

where *χ_eff_* is the effective magnetic susceptibility (the difference in susceptibility between the bead and the fluid), *V_b_* is the bead volume, *B* is the magnetic flux density, and *μ*_0_ is the permeability of the vacuum.

For whole blood in equilibrium with ambient air, the hemoglobin within RBCs exists in the form of diamagnetic oxyhemoglobin, making the RBCs unresponsive to the applied magnetic field. However, for methemoglobinemia, when intracellular oxyhemoglobin is converted to paramagnetic methemoglobin, the RBCs become less diamagnetic than the aqueous medium and migrate as a suspension along the magnetic field gradient [69]. Sun et al. [69] characterized the magnetophoretic mobility and magnetically induced velocity of the oxygenated, deoxygenated, and methemoglobin forms of human RBCs.

Usually, magnetic manipulation and sorting of blood cells and CTCs are based on magnetic particles, making the cells susceptible to magnetic forces [70]. For example, Gómez-Pastora et al. [71] proposed the use of magnetic beads functionalized to capture and separate target pathogens from blood for extracorporeal detoxification. Microfluidic magnetophoretic isolation of target cells spiked in 1 mL of blood at a level of 1000–2000 cells/mL was developed by Li et al. [72].

### 3.3. Optical Methods

#### 3.3.1. Laser Tweezers and Optical Forces

Optical manipulation and the trapping of microparticles with laser tweezers are widely used to characterize blood cells.

In the Mie regime, the scattering (*F_scatt_*) and gradient forces (*F_grad_*) arising from the propagation of a single beam through the dielectric sphere can be expressed as follows [73]:

(28) $$F_{scatt}=\frac{n_{m}P}{c}\left(1+R\cos2\theta_{i}-\frac{T^{2}\left[\cos(2\theta_{i}-2\theta_{rf})+R\cos2\theta_{i}\right]}{1+R^{2}+R\cos2\theta_{rf}}\right),$$

(29) $$F_{grad}=\frac{n_{m}P}{c}\left(R\sin2\theta_{i}-\frac{T^{2}\left[\cos(2\theta_{i}-2\theta_{rf})+R\cos2\theta_{i}\right]}{1+R^{2}+R\cos2\theta_{rf}}\right),$$

where *n_m_* is the refractive index of the medium, *P* is the power of the incident light, *θ_i_* and *θ_rf_* are the angles of incidence and refraction, respectively, *R* and *T* are the Fresnel reflection and transmission coefficients, respectively, and *c* is the speed of light.

The refractions and reflections of a single light beam are shown in Figure 2E(i). The total force acting on the sphere is the sum of the contributions from each beam entering the large numerical aperture objective (Figure 2E(ii)). In the equilibrium position of the cell, the sum of all forces is zero. The scattering and gradient forces depend on the physical properties of the biological cell—that is, the refractive index and radius of the cell, as well as the Fresnel reflection and transmission coefficients. The equilibrium of the optical forces with the electrical, magnetic, and/or hydrodynamic forces makes the determination of many other cell characteristics possible. Optical tweezers have also been used to study the mechanical properties of blood cells. Wottawah et al. [74] described the application of an optical stretcher to determine the viscoelastic moduli of a single suspended cell and cytoskeleton. Several recent reviews have discussed the progress in the use of optical tweezers for blood cell research [75,76,77]. Optical tweezers and optofluidic sorting are also applicable to nanosized particles in the study of biological materials [78].

#### 3.3.2. Image Analysis

Microscopic image acquisition and image analysis are important steps in the study of (bio)particles. One of the more well-established methods for analyzing blood flow images, cell trajectories, and velocity profiles is time-resolved microparticle image velocimetry (μ-PIV) [52,79]. As an alternative to microscopic imaging, Li et al. [80] placed a microfluidic chip directly on a lensless CMOS image sensor (CIS), allowing them to form a diffraction image of an erythrocyte on the CIS. Next, diffraction image processing was used for erythrocyte recognition and posture estimation. In particular, the effect of erythrocyte flipping was analyzed.

### 3.4. Acoustic Radiation Force

The acoustic radiation force (*F_A_*) acting on particles suspended in a fluid medium can be described as [81,82]:

(30) $$F_{A}=-\left(\frac{\pi p_{0}^{2}V_{p}\beta_{f}}{2\lambda }\right)\cdot \left(\frac{5\rho_{p}-\rho_{f}}{2\rho_{p}+\rho_{f}}-\frac{\beta_{p}}{\beta_{f}}\right)\cdot \sin\left(\frac{4\pi }{\lambda }x\right),$$

where *p*_0_ is the pressure amplitude, *V_p_* is the volume of the particle, *λ* is the wavelength of the acoustic wave, *β_f_* and *β_p_* are the compressibilities of the medium and particles, respectively, *ρ_f_* and *ρ_p_* are the corresponding densities, and *x* is the distance from the pressure node.

Based on the physical particle properties, the radiation force moves the particles to either a pressure node or an antinode. The acoustic radiation force equals zero at equilibrium (node or antinode). Using acoustic power to determine the physical properties of blood cells in microfluidic devices is challenging. However, acoustic pressure is widely used for particle separation—for example, in high-throughput separation of platelets from whole blood [82]. Recent advances in acoustic fluid separation of cells and particles have been reviewed by Wu et al. [83].

### 3.5. Mechanical Properties

The mechanical properties of blood cells, particularly erythrocytes, are complex. The inner region of the erythrocyte is a viscous fluid covered by a viscoelastic membrane and a supporting cytoskeleton. A simplified mechanical model assumes that the erythrocyte has the elastic properties of a solid, e.g., tensile and shear deformations, as shown in Figure 2F(i) and (ii), respectively. Occasionally, the model is simplified further to a spring model (Figure 2F(i)), but more often the elastic properties are described by the DI, which is usually the ratio between the major and the minor axes of the cell.

The ability of RBCs to change shape under harsh conditions in microvasculature was investigated by Reichel et al. [41]. RBCs demonstrated tumbling, tank-treading, and parachute motions for different flow regimes and channel dimensions (Figure 2F(iii)). The primary parameters describing the mechanical properties of RBCs were the RBC membrane area and volume, membrane shear modulus and bending rigidity, and volume constraint coefficient.

In concluding this section, it is important to note that the physical properties of blood and blood cells can be determined either by direct or indirect measurements. In the case of direct methods, the measured quantities are either used to calculate physical properties or they are required physical properties. In the case of indirect methods, the microfluidic device must be pre-calibrated using fluids or particles with known physical properties. This approach makes it possible to measure, for example, the transit time of a passing particle through a narrow constriction and convert the passing time to the apparent Young’s modulus. However, the use of indirect methods requires some caution.

## 4. Separation and Sorting

The separation and sorting of blood cells is an essential first step for subsequent identification, enrichment, eventual capture and release, manipulation, processing, and analysis. Cell separation is based on the differences in the physical properties of cells. Consequently, separation provides a qualitative comparison of the physical properties of different cells (e.g., harder or softer, larger or smaller, nDEP or pDEP). Since separation is an essential first step in the aforementioned procedures, there are many studies on the subject. Review articles published between 2019 and 2020 provide insights into the latest advances in the field [84,85,86,87,88,89].

CTCs are extremely rare—that is, of the order of ~1 CTC per 10^6^ WBCs and 10^9^ RBCs in 1 mL blood—making isolation and analysis of CTCs significant technical challenges. Recent progress in the field has been discussed in several reviews previously [33,35,90,91,92,93,94,95]. This section focuses on combining the different forces and techniques within a single microfluidic platform for more efficient cell separation.

### 4.1. Separation by a Combination of Hydrodynamic Forces

**Figure 3:**
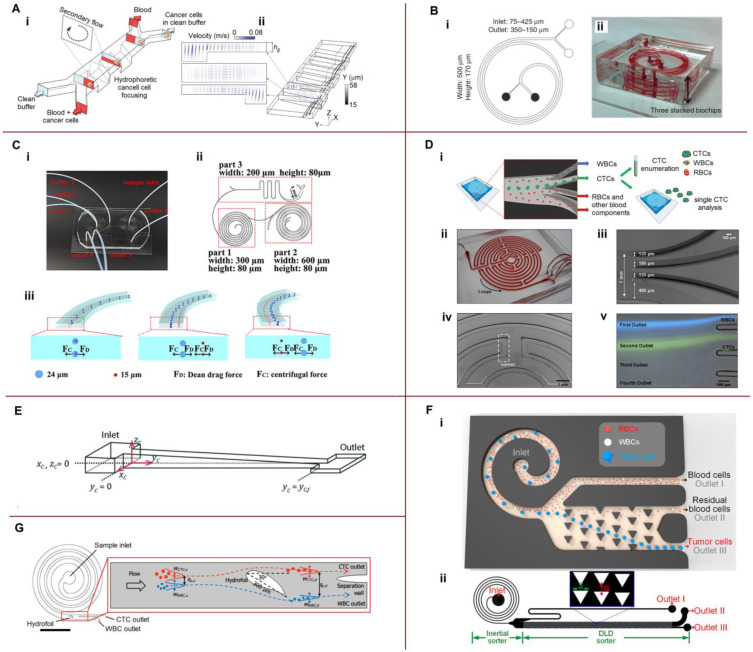
Microfluidic systems for passive separation of cells using a combination of hydrodynamic forces. (**A**) Microfluidic device for continuous sorting and washing of cancer cells from whole blood: (**i**) Inclined trenches in the device create rotating fluid flows. Cancer cells are displaced into a clean buffer. (**ii**) Numerical analysis of three-dimensional rotating flows. Reproduced with permission from [96]. Copyright 2015, The Korean BioChip Society and Springer. (**B**) Three-layered multiplexed spiral biochip: (**i**) CAD drawing. (**ii**) A multiplexed chip obtained by stacking three individual biochips together. Reproduced with permission from [97]. Copyright 2015, Nature America, Inc. (**C**) Cascaded inertial focusing microfluidic channels: (**i**) Device consists of one sample inlet, two buffer inlets, and four outlets. (**ii**) Schematic of chip dimensions. (**iii**) The separation principle and force balance in a zigzag channel. Reproduced with permission from [98]. Copyright 2018, American Chemical Society. (**D**) Microfluidic labyrinth: (**i**) Schematic of labyrinth system. (**ii**) Demonstration of “loops”. (**iii**) Outlet design. (**iv**) Indication of “corners”. (**v**) Separation of labelled WBC/MCF-7 cell line into individual outlets. Reproduced with permission from [99]. Copyright 2017, Elsevier Inc. (**E**) Variable-height microfluidic channel. Reproduced with permission from [100]. Copyright 2020, The Royal Society of Chemistry. (**F**) Two-stage i-DLD device: (**i**) Working principle. (**ii**) CAD drawing illustrating the i-DLD device. Reproduced with permission from [101]. Copyright 2019, American Chemical Society. (**G**) Spiral microchannel combined with a hydrofoil. The length of the scale bar is 3 mm. Reproduced with permission from [102]. Copyright 2020, the authors.

Here, we review the design of novel sorting, separating, and trapping devices that use only combined hydrodynamic forces in a single microfluidic platform. Various microfluidic systems for passive cell separation are shown in Figure 3.

Kim et al. [96] described a new device for the continuous sorting and washing of cancer cells from blood samples using three-dimensional rotating flows and hydrophoretic cells focused in a microchannel with slant trenches (Figure 3A). The cell mixture was introduced using a focusing fluid at a total flow rate of 44 µL/min and a throughput of 4.3 × 105 cells/min. The blood cell rejection efficiency reached 99.3%, and washing efficiency was 99.0%. The generation of vortex flows for microfluidic centrifugation was reviewed by Ahmed et al. [103].

Ultra-fast and label-free isolation of CTCs was achieved by Warkiani et al. [97] (Figure 3B). Their design combined the effects of the inertial lift and Dean drag forces. Ultra-high throughput (7.5 mL in 12.5 min) was obtained by stacking three separate layers together. Abdulla et al. [98] presented a cascaded microfluidic device consisting of two spiral channels and one zigzag channel designed for different fluid fields, including lift, Dean drag, and centrifugal forces (Figure 3C). This device successfully separated RBCs, WBCs, and two different types of tumor cells: the human lung cancer cell line A549 and the human breast cancer cell line MCF-7. The separation occurs because the Dean and centrifugal forces for different particles are balanced at different points (Figure 3C(iii)). An inertial microfluidic centrifuge in combination with a two-stage serpentine channel was developed by Fang et al. [104] for efficient bioparticle extraction. High throughput was achieved by combining four inertial spiral channels connected in cascade with a two-stage serpentine channel. The Dean effect was used by Shen’s group for highly efficient particle separation [105] and flow-rate insensitive blood plasma extraction [106]. They developed an inertial focusing strategy and designed a spiral microchannel with ordered micro-obstacles or microbars.

Lin et al. [99] presented a label-free microfluidic device, Labyrinth, that combined long loops and sharp corners to focus and isolate both CTCs and WBCs at a high throughput of 2.5 mL/min (Figure 3D). A novel and simple method for fabricating tapered glass microchannels of varying heights for the passive trapping of microparticles based on their size and deformability was developed by Mena et al. [100]. The channel height varied linearly from 1 µm to more than 20 µm over a length of approximately 6 cm (Figure 3E). Particles were efficiently and reproducibly separated by size and deformability, including particles with diameters differing by less than 100 nm. Consequently, the proposed device provided qualitative characteristics of cell deformability. Xiang et al. [101] combined inertial microfluidics with deterministic lateral displacement (DLD) and introduced a two-stage i-DLD sorter for precise, continuous, and size-based cell separation with a flow rate of 400 μL/min (Figure 3F).

A spiral microchannel was combined with a hydrofoil by Özkayar et al. [102] to improve the resolution of separation (Figure 3G). They also proposed a two-cycle sample processing technique using the same microfluidic chip. This system had a very high throughput (1.2 mL/min volumetric flow rate) and took less than 9 min for Cycle 1 (10 mL) at the sampling inlet (corresponding to 5 mL of whole blood) and 3 min for Cycle 2. Bazaz et al. [107] investigated zigzag microchannels for rigid inertial separation and enrichment of cells and particles. These devices provide improved particle separation via an induced secondary recirculating (Dean) flow perpendicular to the main flow direction. When comparing soft and rigid zigzag and serpentine inertial microfluidic channels, rigid zigzag channels were found to provide outstanding concentrations of particles or cells, with a concentration efficiency of over 99.99%. Advances and challenges in inertial microfluidics have recently been reviewed by Tang et al. [108] and Xiang et al. [109].

### 4.2. Coupling Hydrophoresis with Other Techniques

**Figure 4:**
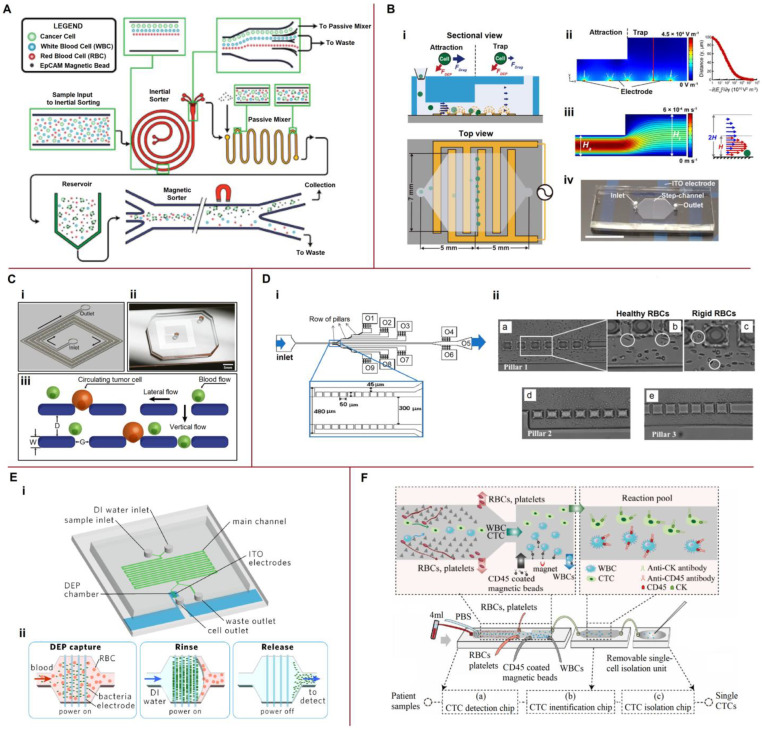
Microfluidic platforms combining hydrophoresis with other techniques. (**A**) Schematic of ultra-specific CTC isolation platform showing inertial sorting, passive mixing with incubation, and magnetic sorting. Reproduced with permission from [110]. Copyright 2016, the authors. (**B**) Design of the step channel and the fabricated device: (**i**) Schematic of the device. (**ii**) Numerical simulation of the electric field. (**iii**) Simulated contours of the flow velocity and streamlines. (**iv**) The fabricated device. The scale bar is 10 mm. Reproduced with permission from [111]. Copyright 2017, the author(s). (**C**) The microfluidic lateral flow filtration (μ-LaFF) chip: (**i**) design and (**ii**) photographic image of the μ-LaFF chip. (**iii**) Lateral and vertical fluidic flows in microchannel and filtration of CTCs. Reproduced with permission from [112]. Copyright 2014 Elsevier B.V. (**D**) Microchannel with geometric pillar variations for cell separation and deformability measurements: (**i**) Microchannel geometry with nine outlets (O1–O9). (**ii**) Visualization of cell deformability. Reproduced with permission from [113]. Copyright 2018, the authors. (**E**) Microfluidic device for desalination and enrichment of bacterial cells: (**i**) Diagram of H-filter and positive dielectrophoresis. (**ii**) pDEP capture, rinse to remove any residues, and DEP release to collect the bacterial cells in the DEP chamber. Reproduced with permission from [114]. Copyright 2018, the author(s). (**F**) Integrated microfluidic platform for CTC isolation and single-cell analysis. Reproduced with permission from [115]. Copyright 2019, International Society for Advancement of Cytometry.

Examples of microfluidic platforms that combine hydrophoresis with other methods are shown in Figure 4. Jack et al. [110] developed a microfluidic device combining inertial microfluidics and immunomagnetism to efficiently isolate CTCs from whole blood with ultra-high purity and high throughput (Figure 4A).

Kim et al. [111] presented a step channel consisting of an attraction zone to control the vertical positions of cells and a trap zone to capture them (Figure 4B). When cells are introduced into the narrow attraction zone, they are attracted to the electrodes on the lower surface of the microfluidic channel through a positive DEP force, although there is a significant drag force pushing the cells through the narrow segment. After sudden channel expansion, the resistance force weakens and the particles are captured by the electrode. Kim et al. [111] noted that integrating cell sorting and trapping into one device could be ideal for developing an automated CTC analysis system. Lee et al. [112] proposed a microfluidic lateral flow filtration (μ-LaFF) chip, wherein lateral flow was combined with the vertical flow into a filter to gently capture CTCs (Figure 4C). Faustino et al. [113] demonstrated the potential of a cross-flow microfluidic device to perform partial passive separation of RBCs based on their deformability (Figure 4D). Moreover, RBC deformability has been assessed using image analysis and spectrophotometry. The left, center, and right arrows in Figure 4D(i) indicate the areas wherein the spacing between the pillars is 17 μm (pillar 1), 16 μm (pillar 2), and 14 μm (pillar 3). Pillars 1, 2, and 3 are shown in Figure 4D(ii). The proposed microfluidic device would require further image analysis automation to assess the deformability of RBCs.

Wang et al. [116] combined the standing surface acoustic waves (SSAWs) and focused the traveling pulsed SAWs (TSAWs) to effectively isolate CTCs from blood samples without using sheath flow. The first pair of straight interdigital transducers was used to generate SSAWs and focus all the particles into the middle of the channel. The second pair of focused interdigital transducers generated the focused TSAWs to push the CTCs away from the blood cells. The separation ratio achieved was 90%. Cai et al. [114] described a microfluidic device that combined H-filter desalination with pDEP to directly enrich bacterial cells from physiological samples with high conductivity and viscosity (Figure 4E). Capture efficiencies of 80.2% were demonstrated by directly capturing bacterial cells in whole human blood after H-filter desalination.

An integrated microfluidic platform for CTC isolation and single-cell analysis was developed by Xu et al. [115]. The platform consists of three parts (Figure 4F). The first part is a CTC detection chip, wherein RBCs, WBCs, and PLTs are depleted by deterministic lateral displacement. The immunomagnetic purifier removes the remaining WBCs. The second part, where CTCs with residual WBCs react with antibodies pre-placed in the chip, identifies the CTCs. The third isolation part was removed from the platform and observed under a microscope, allowing sequential manual cell picking. Using the developed platform, CTCs were detected in 15 peripheral blood samples from 20 patients with cancer, resulting in a 75.0% detection rate. Nguyen and Jen detected CTCs by combining dielectrophoretic manipulation and impedance measurements using circular microelectrodes within a single microfluidic device [117]. The experimental results showed that the impedance sensor was able to detect A549 cells with low cell numbers.

Thus, devices with cascading connections and with multiple manipulating forces are being successfully developed by a number of research groups, which was also confirmed by Cha et al. [118] in a recent review. Although developments in the field of microfluidic separation and sorting of particles have made great progress, challenges remain with respect to increasing device throughput and application in medical practice. Separation of CTCs also remains a challenge, as CTCs are represented by only a few cells per milliliter of blood. Apparently, further work should be focused not only on improving the efficiency of CTC separation and isolation, but also on the subsequent analysis of CTCs on the same microfluidic platform.

## 5. Cytometry and Biophysical Characterization of Blood

Microfluidic cytometry is closely related to hematological tests and has been successfully used for FBC, HCT, and hemoglobin measurements. As mentioned previously, we refer to RBC, WBC, and PLT counts as well as MCV, MCH, MCHC, and HCT as physical properties, which characterize only the blood samples and are independent of the measuring system. However, as a rule, microfluidic cytometers allow additional assessment of some blood biophysical characteristics, which, by our definition, reflect both the properties of the blood and the measuring device. Examples of biophysical characteristics are DI, deformability, AI, and aggregability. Different devices may show different values for the same biophysical characteristics of the same blood sample.

### 5.1. Microfluidic Impedance Cytometry

**Figure 5:**
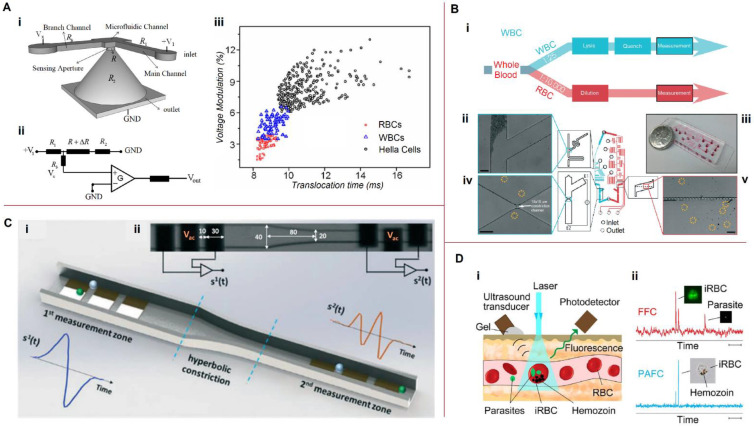
Microfluidic cytometry devices. (**A**) Detection and enumeration of biological cells: (**i**) Design of the micropore. (**ii**) Equivalent electric circuit of the device. (**iii**) Scatter plot of the peak amplitude and translocation time for a mixed cell sample. Reproduced with permission from [119]. Copyright 2014, WILEY-VCH Verlag GmbH & Co. KGaA, Weinheim. (**B**) Key modules for RBC (red) and WBC (blue) dilution, lysis, and filtration: (**i**) Flow through the device for RBC and WBC sample preparation protocols. (**ii**) Module used for lysis of RBCs. (**iii**) Photographic image of the microfluidic device. (**iv**) Module for electrical impedance measurement. (**v**) Filtration modules. Reproduced with permission from [120]. Copyright 2015, The Royal Society of Chemistry. (**C**) Impedance cytometer design for coincidence resolution: (**i**) Schematic of a device with two measuring zones separated by a hyperbolic constriction. (**ii**) Microscopy image of the microfluidic chip. Dimensions are in µm. Reproduced with permission from [121]. Copyright 2020, IEEE. (**D**) In vivo photoacoustic (PA) flow cytometry (PAFC) for malaria detection: (**i**) Optical schematic of integrated PA and fluorescence flow cytometry. (**ii**) Fluorescence and PA signals from circulating malaria parasites expressing green fluorescence protein and iRBCs with absorbing hemozoin, respectively. Reproduced with permission from [122]. Copyright 2016, International Society for Advancement of Cytometry.

Coulter-type devices based on the principle of electric-sensing zones have been widely used for cell analysis on microfluidic platforms. Samples of microfluidic cytometry devices are shown in Figure 5.

Castillo-Fernandez et al. [123] used a simple microfluidic chip consisting of a single microchannel with coplanar perpendicular electrodes. However, they developed an electronic system that allowed the counting rate to increase to approximately 400 cells/s, and the cell sizes were determined based on the voltage amplitude and transition time. Guo et al. [119] presented a micropore-based resistive cytometer for the detection and enumeration of biological cells, wherein particles were released from an upper reservoir and transported through a small sensing aperture employing a pressure-driven flow (Figure 5A(i)). If a single non-conductive particle blocked the micropore, the electrical resistance of the pore increased significantly, increasing the bias voltage, *V_x_*. The equivalent electrical circuit is shown in Figure 5A(ii). The combination of pulse peaks and translocation times allows the tumor cells to be distinguished from blood cells. A mixture of RBCs (1000 cells/mL), WBCs (1000 cells/mL), and HeLa cells (3000 cells/mL) was passed through the device for quantitative evaluation (Figure 5A(iii)). Constriction-based microfluidics can provide information on mechanical properties and cell sizes by considering their transit times through the deformation channel.

On-chip processing of whole blood samples (dilution, lysis, and filtration) and downstream single-cell measurements were fully integrated in a single device by Nguyen et al. [120]. Their device consists of two parallel modules for processing RBCs and WBCs, and subsequent electrical measurements to determine the RBC and WBC parameters (Figure 5B). Coulter counters were used for downstream electrical enumeration and continuous flow analysis after sample preparation. Sinusoidal voltages at multiple frequencies (10, 100, 400, and 990 kHz at 500 mV_pp_) were applied through Ag/AgCl electrodes for electrical impedance measurements. The mean corpuscular volume was quantified by determining the mean value of the fitted Gaussian curve. Ghassemi et al. [124] proposed a system consisting of a constriction-based microfluidic sensor with embedded electrodes to detect and enumerate cancer cells in the blood. One electrode was placed near the inlet of the microfluidic channel; the other electrode was placed near the outlet. The magnitude and phase of the impedance were measured at multiple frequencies as the cells travelled through the channel. The impedance peaks represent the contribution of cell electrical properties, and the peak width represents the cell transit time dependent on cell deformability, both dependent on cell size. The chip had a 1 μL/min throughput and was designed for post-enrichment label-free enumeration and CTC characterization. However, two or more particles could sometimes pass through the sensitive area at approximately the same time, causing errors in cell counting and identification. Caselli et al. [121] proposed a strategy for coincidence resolution using microfluidic impedance cytometry. They used two successive sensing zones instead of one (Figure 5C) and applied a Bayesian approach to decompose the signals generated by the coincident particles into individual contributions. The device had a throughput of approximately 2500 particles/s for RBC analysis.

Reale et al. [125] developed an innovative impedance cytometer to measure the cross-sectional position of individual cells flowing in a microchannel, where the lateral position was measured using a four-electrode configuration and vertical position was measured using a five-electrode configuration. De Bruijn et al. [126] developed a cytometer with coplanar electrodes, which was easy and cheap to fabricate yet sensitive to positional differences between the passing particles. They also presented a particle-height compensation method that employed the dependence of the measured electrical opacity on particle height and determined particle sizes. Serhatlioglu et al. [127] proposed the suspension of RBCs in a polyethylene oxide-based viscoelastic solution for impedance cytometry. The solution provided uniform orientation and single-train focusing of non-spherical RBCs in the sensitive zone. To analyze rare CTCs, enrichment is necessary before counting to remove large numbers of interfering blood cells. A microfluidic-based Coulter counter (μCC) was designed by Kong et al. [128] for rare cell counting without labelling. The μCC was integrated into the CTC enrichment chip to avoid the pre-treatment of whole blood that usually led to the loss of rare cells. Future work will focus on developing an integrated platform for CTC analysis.

### 5.2. Photoacoustic Flow Cytometry

Photoacoustic flow cytometry, based on the detection of generated acoustic waves resulting from thermal expansion of tissues illuminated by short laser pulses, has been a fundamental tool for discoveries in cell biology and disease diagnosis for many years.

Cai et al. [122] applied in vivo photoacoustic (PA) flow cytometry (PAFC) for the early diagnosis of malaria through label-free detection of hemozoin produced by malaria parasites in infected RBCs (iRBCs) as a high-contrast PA agent (Figure 5D). The PAFC performance was verified in vitro using a 50 μm capillary tube with a flow velocity of 1 cm/s as a vessel phantom. Malaria-infected mice were used for in vivo detection of iRBCs using PAFC. The results showed that linear PAFC had the potential for non-invasive malaria diagnosis for an extremely low level of parasitemia (0.0000001%)—that is, ~10^3^ times better than existing tests. Gnyawali et al. [129] developed a label-free microfluidic acoustic flow cytometer combined with ultra-high-frequency ultrasound (UHF US) backscatter and laser-induced PA waves from individual cells and particles flowing through a microfluidic device. Their device demonstrated a throughput of 150 cells/min. Zhao et al. [130] developed an on-chip PA flow cytometry system using a simple microfluidic chip combined with a PA imaging system to count and characterize up to 60 erythrocytes per second. They also demonstrated that the PA signals of erythrocytes flowing in a microfluidic device can be directly used to obtain the osmolarity conditions of the surrounding medium.

### 5.3. Optical Characterization of Blood

**Figure 6:**
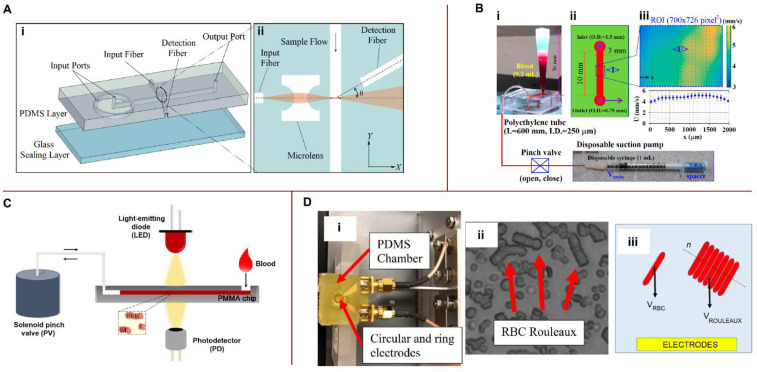
Systems for optical characterization of blood properties. (**A**) On-chip optical system for counting RBCs and PLTs: (**i**) Assembly of the microfluidic chip. (**ii**) Schematic of the optical systems. (**iii**) Microscopic image of on-chip integrated optics. Reproduced with permission from [131]. Copyright 2016, the author(s). (**B**) Measurement of RBC aggregation under periodic on–off blood delivery: (**i**) Schematic of the experimental setup: a suction pump and a microfluidic device with a pipette tip. (**ii**) Microfluidic channel. (**iii**) Averaged blood velocity and averaged image intensity. Reproduced with permission from [79]. Copyright 2017, the author. (**C**) Microfluidic system used for ESR measurement: Solenoid pinch valve used to disaggregate RBCs. Reproduced with permission from [132]. Copyright 2017, Springer-Verlag Berlin Heidelberg. (**D**) EIS of blood during sedimentation: (**i**) Photographic image of the experimental setup. (**ii**) Microscopic image of RBC rouleaux formation. (**iii**) Schematic cross-sectional view of the chamber. Reproduced with permission from [133]. Copyright 2021, the author(s).

Optical equipment is widely used for blood characterization on microfluidic platforms. Systems for optical characterization of blood properties are shown in Figure 6.

#### 5.3.1. Optical Cell Counting

Zhao et al. [131] designed a microfluidic cytometer with integrated on-chip optical systems for RBC and PLT counts (Figure 6A). The input fiber guided the excitation light perpendicular to the microchannel, with the detection fiber being oriented at a small angle relative to the incident light. Forward scatter signals arising from the particles were delivered to the photodetector, with the intensity of the forward scatter signals—measured in volts—providing information on particle size. The resolution performance of the microfluidic cytometer was acceptable for resolving the RBC and PLT sizes from the detector signals. Park et al. [134] presented holographic cytometry as a quantitative phase imaging system to acquire a large number of images of cells in flow by incorporating customized microfluidic chips and stroboscopic illumination. Holographic cytology images provide measurements of multiple physical traits of the cells, including optical volume and area. Peng et al. [135] designed and tested a prototype microfluidic cytometer that integrates a 3D hydrodynamic focusing system and an on-chip optical system for counting and classifying WBCs. The excitation optical system is composed of a laser diode, a fiber coupler, an excitation fiber, and an air microlens. The optical signals, including small-angle forward scatter, large-angle side scatter, and fluorescence, are detected by a photodiode. By analyzing the optical signals, WBCs are identified and classified into four categories: eosinophils, monocytes, neutrophils, and lymphocytes.

#### 5.3.2. Erythrocyte Aggregation and Sedimentation Rates

Kaliviotis et al. [136] analyzed the local aggregation of RBCs in microchannels using bright-field microscopic imaging. The particle image velocity (PIV) technique was applied to quantify the velocity fields and local aggregation of human RBCs in the microchannel simultaneously. They found that the level of aggregation and local hematocrit correlated well with local variations in velocity in both the bulk flow and wall regions. These results could help characterize the structural properties of whole blood in a microchannel. While Kaliviotis et al. [136] continuously mixed blood in the inlet reservoir with a magnetic stirring bar to minimize the aggregation of RBCs, Kang examined the RBC aggregation and erythrocyte sedimentation rate (ESR) at extremely low shear rates in a microfluidic channel [79]. A schematic of the microfluidic-based technique proposed by Kang is shown in Figure 6B [79]. Sedimentation of the blood sample (~0.2 mL) was carried out in a conical pipette tip. Periodic on-off switching of the blood supply to the microfluidic channel was performed using a disposable suction pump and pinch valve (open–closed) (Figure 6B(i)). Microscopic images were captured consecutively in the region of interest using a high-speed camera to monitor blood velocity and image intensity (Figure 6B(ii)). The averaged blood velocity and image intensity were calculated using μ-PIV and digital image processing, respectively (Figure 6B(iii)). Changes in light intensity were used to determine the AI and erythrocyte sedimentation rate aggregation index (EAI).

The mechanism of RBC aggregation in microfluidic channels was examined by Isiksacan et al. [132]. Their measurement system consisted of a blood-filled polymethyl methacrylate microfluidic chip, a near-infrared LED, a photodetector, and a solenoid pinch valve (Figure 6C). The solenoid pinch valve was operated for 15 s to generate a continuous reciprocating movement of the blood sample and disaggregate the RBCs. In this instance—that is, with strongly disaggregated RBCs—the intensity of the transmitted optical signal measured by the photodetector was at its lowest value. When the pinch valve was closed, the RBCs began to aggregate. The intensity increased until complete RBC aggregation occurred, after which the intensity remained constant. A correlation was found between changes in light intensity, aggregation dynamics, and ESR. This correlation was applied to shorten the ESR time using RBC aggregation. In their recent work, Isiksacan et al. [137] implemented an updated method to analyze multiple parameters of blood from a 50 μL sample in 3 min. In the modified design, the solenoid pinch valve was replaced with a pump that mechanically moved the actuator pin back and forth at a frequency of 0.5 s^−1^ for a duration of 14 s. Optical transmission through the blood sample was recorded during flow and at stasis for both healthy RBCs and RBCs with stiffened cell membranes suspended in three different media. The influence of medium viscosity, medium elasticity, RBC stiffness, and RBC aggregability on the optical transmission signals was revealed.

Changes in the β-dispersion of blood during sedimentation were studied by Sabuncu et al. [133] Blood samples were placed in a polydimethylsiloxane (PDMS) chamber with circular and ring electrodes at the bottom. Their electrical impedance was measured periodically using an impedance analyzer as RBCs formed rouleaux and sediment in the PDMS chamber (Figure 6D). The effective radius of aggregated RBCs during sedimentation (*R_agg_*) was described in the work of Zhbanov and Yang [138]:

(31) $$R_{agg}(t)=r+v_{agg}\int_{0}^{t}\frac{1}{\left(1-\varphi \right)}\exp\left[-{\left(\frac{t^{*}}{t_{agg}}\right)}^{2}\right]dt^{*},$$

where *ν_agg_* and *t_agg_* are empirical coefficients characterizing the rate of aggregate formation (*m*/*s*) and the characteristic time of aggregation (s), respectively, and *r* is the radius of a single RBC. The local volume fraction of RBCs (*ϕ*) is time-dependent. For dynamic modelling of RBC sedimentation and changes in the volume fraction, Sabuncu et al. [133] implemented a published algorithm [138]. Consequently, they found the aggregation parameters *ν_agg_* and *t_agg_* and the membrane capacitance *C_mem_* to model the time response of aggregating RBCs and describe the RBC sedimentation kinetics.

#### 5.3.3. Mechanical Properties, Deformability

**Figure 7:**
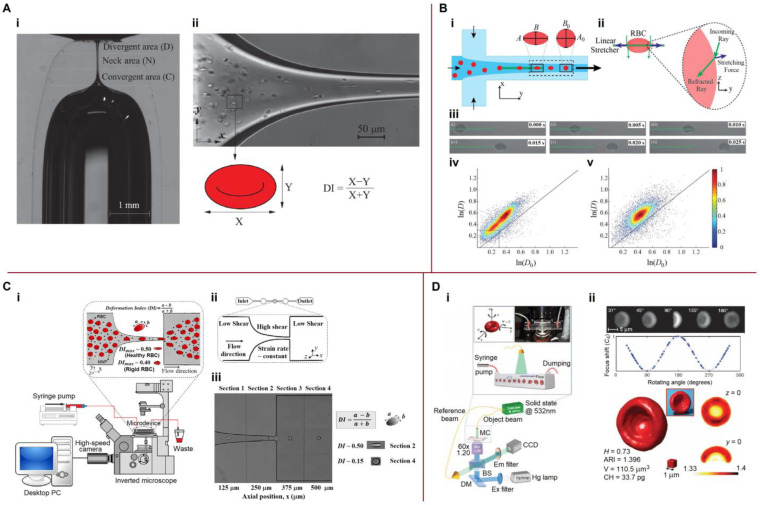
Systems for characterizing the deformability of biological cells. (**A**) Cell deformability in micronozzle: (**i**) Whole shape of a glass micronozzle with a 37 μm neck diameter. (**ii**) Healthy RBCs flowing through a micronozzle. (**iii**) DI as a function of the section diameter for healthy (normal bars) and chemically treated (meshed bars) human RBCs. Reproduced with permission from [139]. Copyright 2019, IOP Publishing Ltd. (**B**) Linear optical stretcher: (**i**) Hydrodynamic focusing of cell and optical stretching. Cells observed in a rectangular region of interest (125 × 15 μm). (**ii**) Optical stretching forces. Stretched aspect ratio is *D* = (*A*/*B*), and the relaxed aspect ratio is *D*_0_ = (*A*_0_/*B*_0_). (**iii**) RBC stretch and relaxation cycle. The green bar shows the position of the linear optical stretcher. (**iv**) Cell aspect ratio for pure RBCs. (**v**) RBCs treated with 3.8 mM diamide. Reproduced with permission from [140]. Copyright 2015, International Society for Advancement of Cytometry. (**C**) Microfluidic studies of the RBCs in contact with MNPs: (**i**) Schematic view of the experimental setup. (**ii**) Microchannel device geometry with the zoom of the hyperbolic channel. (**iii**) Image and DI of healthy RBCs flowing along the axial position of a microchannel with a hyperbolic-shaped contraction followed by a sudden expansion. Reproduced with permission from [141]. Copyright 2016, Springer Science + Business Media Dordrecht. (**D**) Rolling tomographic phase microscopy (R-TPM): (**i**) Working principles. (**ii**) R-TPM results. One-side concavity of RBC. Reproduced with permission from [142]. Copyright 2017, the author(s).

Systems for characterizing the deformability of biological cells are shown in Figure 7. Rubio et al. [139] proposed using a cylindrical glass micronozzle to study cell deformability (Figure 7A). An inverted microscope, halogen light source, and high-speed CMOS camera were used to obtain digital images of the cells. The flow through the micronozzle was experimentally characterized by numerical modelling. For the mechanical characterization of blood cells, Roth et al. [140] used a linear diode bar source in a microfluidic channel, where cells were hydrodynamically focused into the optical stretcher (Figure 7B). The free-stream cell velocity was approximately 2000 μm/s. When the cells reached the stretching region, opposing optical forces created by refracting the highly focused laser light at the cell membrane deformed each cell in the stream. A scatter plot of the stretched aspect ratios vs. the relaxed aspect ratios represents the elastic properties of the cells (Figure 7B(iv,v). Rodrigues et al. [141] studied the deformability of human RBCs upon contact with magnetic nanoparticles (MNPs) with a diameter of approximately 18 nm, suitable for theranostic applications (i.e., the simultaneous diagnosis and therapy of cancer). The microchannel geometry was a hyperbolic-shaped contraction followed by a sudden expansion. In the hyperbolic section, the velocity increased almost linearly, and the strain rate was maintained at an approximately constant level. The microscopy system consisted of an inverted microscope combined with a high-speed camera (Figure 7C), making the use of DI as an indicator of healthy RBCs and assessment of the physiological effects of MNPs on RBC membranes possible.

Label-free tomographic phase microscopy (TPM) was used by Merola et al. [142] for high-throughput single-cell analysis (Figure 7D). The use of a complete angular revolution of cells in the microscope’s field of view and the application of the tomographic reconstruction algorithm allowed the complete characterization of cells in terms of several metrics, including 3D morphology, corpuscular hemoglobin (CH), volume (V), and average refractive index (ARI) (Figure 7D(ii)). This approach was named rolling-TPM (R-TPM). Besedina et al. [143] fabricated a microfluidic device that simulated a capillary network to characterize erythrocyte circulation under oxidative stress. Data for the analysis of erythrocyte transport in the microchannels were recorded using a high-speed camera and microscope. The recorded videos were processed in MATLAB for analysis of the transient velocity and deformation of erythrocytes under various oxidative stresses. The specific topics in microfluidic deformability flow cytometry have been reviewed by Chen et al. [144].

To conclude this section, it can be noted that in many studies only the biophysical characteristics of blood are determined. Apparently, more efforts should be focused on determining the real physical properties that do not depend on the measuring system.

## 6. Physical Properties of Blood and Blood Cells

In this section, we first discuss the methods used to obtain the dielectric properties of blood and blood cells. Then, we present an overview of the available methods for analyzing ion concentration and mobility. Next, we consider the measurement of the cells’ mechanical properties and the viscosity of blood and intracellular fluid. Finally, we review multifunctional and multiparameter devices.

### 6.1. Dielectric Properties, Ion Concentration and Ion Mobility

**Figure 8:**
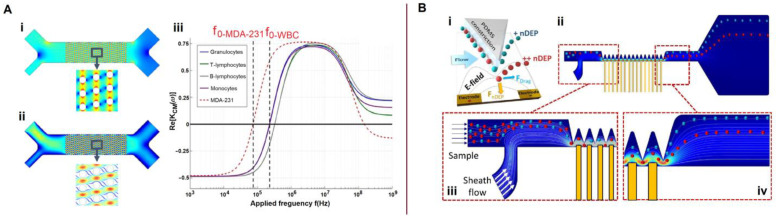
Microfluidic systems for measuring the dielectric properties of blood and blood cells. (**A**) Coupled DLD-DEP device: (**i**) Electric field norm (*V*/*m*). (**ii**) Velocity field (*m*/*s*). (**iii**) The real part of Clausius–Mossotti factor for granulocytes, T-lymphocytes, B-lymphocytes, monocytes, and MDA−231. Reproduced with permission from [151]. Copyright 2019, WILEY-VCH Verlag GmbH & Co. KGaA, Weinheim. (**B**) The working principle of a dynamic dielectrophoresis (Dy-DEP) device: (**i**) Balancing nDEP and drag forces. (**ii**) Schematic of chip design. (**iii**) Focusing effect of the sheath flow. (**iv**) Cells with different DEP characteristics follow different streamlines. Reproduced with permission from [152]. Copyright 2020, Springer-Verlag GmbH Germany, part of Springer Nature.

Bao et al. [145] assessed the degree of a burn injury from the electrical impedance characteristics of blood with different volume proportions of RBCs and heated RBCs. Benhal et al. [68] applied electrorotation to study the dependence of the rotation rate of bovine oocytes (~120 μm diameter) on the conductivity of the buffer medium and the applied frequency in the experiment. The maximum observed rotation rate was approximately 140°⋅s^−1^. In recent years, the EIS method has been successfully applied for blood glucose detection [146,147,148,149,150]. Microfluidic systems for measuring the dielectric properties of blood and blood cells are shown in Figure 8.

Aghaamoo et al. [151] proposed a combination of deterministic lateral displacement (DLD) and insulator-based DEP (iDEP) for the continuous separation of CTCs from peripheral blood cells with high throughput and efficiency (Figure 8A). These methods rely on physical markers, such as size and dielectric properties, to differentiate between different cell types. An electric field in the same direction as microfluidic flow was applied between the inlet and the outlet of the device. The numerical calculations (Figure 8A(i,ii)) were obtained using a buffer inlet velocity of 200 µm/s, blood inlet velocity of 100 µm/s, applied inlet voltage of 10 V, and arrays of DLD microposts with the row shift fraction *ε* = 1/5. In Figure 8A(iii), f_0,MDA−231_ is the crossover frequency of the MDA-231 CTC, and f_0,WBC_ is the crossover frequency of granulocytes, as representatives of WBCs. The frequency-based analysis for various electric field strengths allowed the determination of crossover frequencies. However, the device did not have an automatic crossover frequency-detection function. Dielectrophoretic frequency dispersion is significant for the phenotypic quantification of cells. In a recent study, Torres-Castro et al. [152] developed a dynamic DEP (Dy-DEP) method for the high-throughput measurement of the dielectric dispersion of single cells to stratify the phenotypic heterogeneity of a particular sample based on their DEP crossover frequency (Figure 8B). In the microfluidic device, localized constrictions in the microchannel were used to amplify the field generated by adjacent planar electrodes. Consequently, by analyzing the deviation of the flow streamlines, determining the level of DEP and crossover frequency and distinguishing between the pDEP and nDEP for each particle is possible.

EIS methods for measuring the properties of blood have continuously improved. Pradhan et al. [153] optimized and fabricated four electrode-based sensors for blood impedance measurements. The use of a four-electrode technique with gold electrodes—instead of conventional two- or three-electrode technologies—made it possible to minimize the parasitic capacitance of the double layer created by the polarization effect at the electrode–electrolyte interface. Vital information about the cell could be obtained by rotating and imaging the cell at different angles. Puttaswamy et al. [154] used two parallel sidewall 3D electrodes to generate a dielectrophoretic force and trap RBCs inside the capturing chambers of the microfluidic device, while the hydrodynamic force generated precise rotation of the cells inside the chamber. They also demonstrated the use of a biochip for preliminary concentration and rotation of RBC clusters in 3D. A proportional linear change in the rotation rate of erythrocyte clusters was observed depending on the applied frequency and voltage. The specific topic of microfluidic systems based on low-voltage DEP has been recently reviewed by Ramirez-Murillo et al. [155].

Yalçın et al. [156] modified an ion-release-based impedance spectroscopy method. The average total ion concentration per cell was determined by measuring the variations in solution conductance after the lysis of a population with a known initial cell count. The average radius and volume were previously measured for each type of lysed cell. The calculated ion concentrations for K562/wt, K562/imaR, CCRF-CEM/wt, and CCRF-CEM/doxR cells were approximately 182, 327, 162, and 194 mM, respectively. The electrokinetic and dielectrophoretic mobilities of each *Listeria monocytogenes* serovar were determined by Crowther et al. [63] using a combination of electrokinetic velocity and dielectrophoretic trapping assessments. The basic principles of electrophoretic mobility are briefly described in Section 3.2. The electrokinetic mobility was found to be 19 ± 0.7, 17 ± 0.7, and 9.2 ± 0.3 × 10^−9^ m^2^V^−1^s^−1^ for the *L. monocytogenes serovars* 1/2a, 1/2b, and 4b, respectively.

### 6.2. Elastic Characteristics of Cells

**Figure 9:**
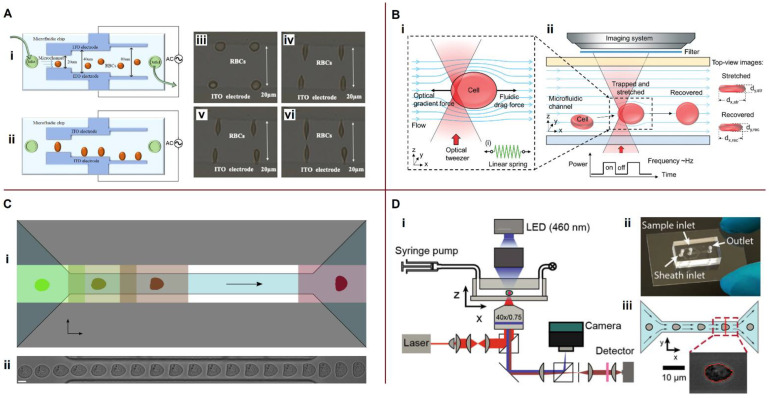
Microfluidic platforms for measuring the mechanical properties of blood cells. (**A**) Deformation of RBCs between parallel ladder electrodes under different voltages: (**i**) RBCs fill the microchannel. (**ii**) RBCs deformed under constant DEP stress. (**iii**) RBCs captured and then deformed under: (**iv**) 2 V_pp_, (**v**) 4 V_pp_, and (**vi**) 6 V_pp_. Reproduced with permission from [157]. Copyright 2019, Springer-Verlag London Ltd., part of Springer Nature. (**B**) Optofluidic “tweeze-and-drag” cell stretcher: (**i**) Working principle diagram. Inset: a linear spring model of a cell under a stretching force. (**ii**) Top view of stretched and reconstructed cells and their x- and y-dimensions. Reproduced with permission from [158]. Copyright 2020, The Royal Society of Chemistry. (**C**) Dynamic real-time deformability cytometry (dRT-DC): (**i**) Experimental setup and region of interest at selected time points t_1_, t_2_, t_3_, and t_n_. (**ii**) Time series of a cell moving along a channel (30 × 30 μm cross section, 300 μm length). Reproduced with permission from [159]. Copyright 2019, the author(s). (**D**) Real-time fluorescence and deformability cytometry (RT-FDC): (**i**) Experimental setup. (**ii**) Photographic image of a PDMS microfluidic chip. (**iii**) Representative image of a cell passing through a narrow constriction. Reproduced with permission from [160]. Copyright 2019, Springer Science + Business Media, LLC, part of Springer Nature.

Alterations in RBC mechanical properties and shape are often associated with blood diseases and disorders, such as sickle cell anemia, diabetes mellitus, malaria, etc. [41], which emphasizes the importance of studying this problem. Examples of microfluidic platforms for measuring the mechanical properties of blood cells are shown in Figure 9.

Li et al. [157] presented a new method for the mechanical characterization of RBCs based on a DEP microfluidic system integrated with parallel ladder electrodes (Figure 9A). Initially, the RBCs flowed in the microchannel from the inlet to the outlet. When the flow was stopped, RBCs were attached to the edges of the electrodes and stretched by DEP forces. A change in the gaps between the electrodes resulted in a change in the DEP forces. A CCD camera was used to capture the stretching and relaxation of erythrocytes. The DEP forces applied to the cells were calculated using Equation (19). The gradient of the electric field strength squared ($\nabla E_{0}^{2}$) was modelled using COMSOL Multiphysics. The strain (*γ*) was expressed as *γ* = (*a*−*a*_0_)/*a*_0_, where *a* denotes the length of the long axis of the stretched elliptical cell and *a*_0_ denotes the original length. As a result, a change in the RBC strain was observed, dependent on the DEP force generated by the different electrodes. Yao et al. [158] combined optical and microfluidic techniques and demonstrated an optofluidic cell stretcher based on a “tweeze-and-drag” mechanism using a periodically chopped, tightly focused laser beam as an optical tweezer to temporarily capture the cell and a drag force to stretch it in the microfluidic channel in the flow direction (Figure 9B). Recorded video clips were processed using MATLAB’s toolbox software. The mechanical properties of RBCs can be described using a linear relationship between the stretching force and cell elongation in the *x*-direction (Δ*d_x_*): *F* = *k*Δ*d_x_*, where *k* is the spring constant. Consequently, the fluidic drag force exerted on the trapped cells was estimated using a model based on Stokes’ law for a thin circular disk shape: *F* = 32*ηrv*/3, where *η* is the dynamic viscosity of the fluid, *r* is the radius of the disk, and *v* is the relative speed between the trapped cells and the flow. The obtained spring constant of RBCs was ~14.9 μN m^−1^.

Using real-time deformability cytometry (RT-DC), Reichel et al. [41] combined the simulation and experimental studies of RBC shapes and dynamics in microchannels. They described changes in the shapes of RBCs (tumbling, tank walking, and parachute) as a function of shear rate in terms of a probability density distribution. Elitas and Sengul [64] studied the dielectric movement and deformation of U937 monocytes and U937-differentiated macrophages in a low-conductivity medium within a 3D carbon-electrode array. For these cells, they estimated the crossover frequency, dielectrophoretic DI, dielectric mobility, and membrane relaxation time. The resulting images of cell movements were analyzed manually using open-access ImageJ software. The crossover frequency was defined as the cessation of particle motion, which is a characteristic of particles. Monocytes exhibited a crossover frequency of approximately 150 kHz, while the macrophage crossover frequency was below 50 kHz. DI (the ratio between the major and minor axes of the cell) was calculated by manually measuring the height and width of every single cell. U937 monocytes exhibited maximum and minimum DIs at 400 kHz and 1 MHz, respectively, at 20 V_pp_. The dielectric mobility and membrane relaxation time were calculated using Equations (22) and (23) based on experimental data.

Fregin et al. [159] introduced dynamic real-time deformability cytometry (dRT-DC) for single-cell rheological measurements in heterogeneous samples at up to 100 cells/s (Figure 9C). They used Fourier expansions to disentangle a cell’s response to complex hydrodynamic stress distributions and determine viscoelastic parameters independent of cell shape. The field of view (1280 × 80 pixels) covered the entire length and width of the microfluidic channel, including the inlet and outlet areas, where the camera captured images at frame rates of between 2000 and 6000 fps. For RBCs, Fregin et al. [159] determined an apparent Young’s modulus of *E* = 0.10 ± 0.01 kPa, while granulocytes (*E* = 0.43 ±0.05 kPa) and peripheral blood mononuclear cells (PBMCs) (*E* = 0.62 ± 0.04 kPa) were significantly stiffer. They also found that the apparent viscosities exhibited statistically significant differences at *η* = 0.24 ± 0.18 Pa⋅s for erythrocytes, *η* = 5.22 ± 0.34 Pa⋅s for granulocytes, and *η* = 8.70 ± 0.45 Pa⋅s for PBMCs.

Feng et al. [161] proposed camera-free intrinsic mechanical cytometry (CFIMC) for on-the-fly measurement of the Young’s modulus (*E*) and fluidity (*β*) of individual cells. First, they recorded time-resolved microscopic images of squeezing beads at different points in time using a high-speed camera. The elastic moduli of these beads were then measured by atomic force microscopy to calibrate the measuring system. The mechanical parameters were found to be *E* = 0.615 ± 0.159, 1.317 ± 0.410, and 0.388 ± 0.093 kPa, and *β* = 0.323 ± 0.084, 0.281 ± 0.078, and 0.367 ± 0.081 for untreated, fixed, and CytoB-treated MDA-MB-231 cells, respectively. Jacobi et al. [160] focused on the implementation of a rapid method suitable for clinical requirements called real-time deformability cytometry (RT-DC) to study the mechanical properties of cells. The method was based on the analysis of cell shape as a cell passed through a narrow constriction. A bright-field microscope equipped with a high-speed camera was used to obtain images. The deformation parameter (*D_c_*) was defined as follows:

(32) $$D_{c}=1-\frac{2\sqrt{\pi A_{c}}}{P_{c}},$$

where *A_c_* is the projected surface area and *P_c_* is the cell perimeter.

An analytical model, which has recently been extended numerically, allows the extraction of Young’s modulus (*E*) as a material parameter based on cell deformation and cell size [162,163]. Images were analyzed in real-time at a rate of 1000 cells/s. Jacobi et al. [160] determined that CD34^+^ cells have a mean size of 61.1 ± 1.3 μm^2^ and an elastic modulus of approximately 0.94 ± 0.24 kPa. Compared to the CD34^+^ fraction, CD34^−^ cells have a higher elastic modulus (similar to lymphocytes with 1.07 ± 0.38 kPa) or a lower elastic modulus (similar to granulocytes/monocytes with 0.908 ± 0.14 kPa) and exhibit a more heterogeneous size distribution.

### 6.3. Viscosity

The plasma viscosity test belongs to the inflammatory panel of basic blood tests and provides a measure of the acute phase response. As mentioned previously, many characteristics of blood in microfluidics are assessed via their correlations with other parameters.

Sometimes, quantities do not have a precise physical definition and/or depend on the parameters of the device with which they are measured, e.g., deformability, aggregation index, transit time, etc. This approach was applied by Kucukal et al. [164] to combine a microfluidic assay with a microparticle image velocimetry technique (μ-PIV) for the integrated in vitro assessment of whole blood viscosity and RBC adhesion. Whole blood viscosity was correlated with RBC count and hematocrit in subjects with sickle cell disease. A logarithmic correlation was found between the mean flow velocity and whole blood viscosity, measured using a commercially available piston-type viscometer. However, microfluidic methods can directly measure liquid viscosity as a physical quantity without requiring any other properties of the liquid. Several recent studies have focused on the apparent viscosity of whole blood in microfluidic channels [165,166,167,168].

Solomon et al. [169] proposed an interesting method for measuring blood viscosity. The basic principle was as follows: blood entered the microchannel inlet under controlled pressure, the microchannel outlet was connected to a horizontal capillary tube whose end was open, and the air pressure was maintained. The motion of the air–blood interface in the capillary tube was recorded using a smartphone camera. The speed of the moving interface made the determination of the flow rate and the calculation of viscosity using Equation (8) possible. The surface tension at the air–blood interface was considered to be a parasitic effect. The developed devices made the measurement of the viscosity of any liquid, not only whole blood but also pure plasma, possible. Nevertheless, combining the separation of plasma from whole blood and measurement of plasma viscosity in a single device is challenging. However, to characterize blood and blood cells, viscosity measurements can be combined with other technologies in one platform. A microviscometer for measuring shear-varying blood viscosity over a wide range of shear rates was developed by Kim et al. [170]. Later, this viscometer was integrated with other devices in a microfluidic platform [171]. The influence of the mechanical properties of an erythrocyte membrane on the viscosity of whole blood has been recently reviewed by Trejo-Soto et al. [172].

### 6.4. Multidisciplinary Methods, Multiparameter Devices

#### 6.4.1. Multifunctional Microfluidic Platforms

A microfluidic device for separating a certain number of RBCs from blood and then measuring the deformability of individual RBCs downstream in one platform was presented by Pinho et al. [173]. In the separation module of the device, constriction followed by sudden expansion was used to separate the plasma with a low concentration of RBCs from whole blood. In the deformation measurement module of the device, the subsequent constriction was used to obtain images and determine the RBC DI. Image analysis techniques allowed the automatic measurement of the cell-free layer thickness before and after artificial narrowing.

King et al. [174] demonstrated a multidisciplinary approach for the identification and characterization of single CTCs and CTC aggregates from the blood of a patient with breast cancer. Non-interferometric quantitative phase microscopy (NIQPM) imaging was performed on both images to quantify the subcellular organization of the dry mass density of CTCs and cultured tumor cells, respectively. The authors investigated the dependence of CTC vascular margination on single CTCs and CTC aggregate morphology and stiffness using a numerical model coupling elastic collisions between RBCs and CTCs. NIQPM imagery revealed the geometric (area, perimeter, aspect ratio, eccentricity) and subcellular density organization (mass, mean density, fluctuations about the mean, coefficient of variation) of single CTCs and CTC aggregates.

Liang et al. [175,176] investigated the translational and self-rotational motion of Raji cells and RBCs in an optically induced non-rotating electric field. A series of elapsed time images of the different translation and rotational responses of the cells were used to characterize them. The Raji cells’ self-rotation rate reached a maximum value of 147.06 ± 8.6 rpm, and the rate of self-rotation of RBCs reached a maximum value of 40.75 ± 4.73 rpm at a frequency of 60 kHz in both cases. For translational motion, the crossover frequency (*f_1_*) can be approximated as:

(33) $$f_{1}=\frac{\sqrt{2}\sigma_{f}}{2\pi rC_{m}},$$

where *C_m_* is the membrane capacitance of the cells, *σ_f_* is the conductivity of the surrounding fluid, and *r* is the cell radius. The crossover frequency of Raji cells was assessed by observing their translational behavior. The membrane capacitances were estimated to range from 7.90 × 10^−3^ ± 0.27 × 10^−3^ F/m^2^ to 9.40 × 10^−3^ ± 0.34 × 10^−3^ F/m^2^.

**Figure 10:**
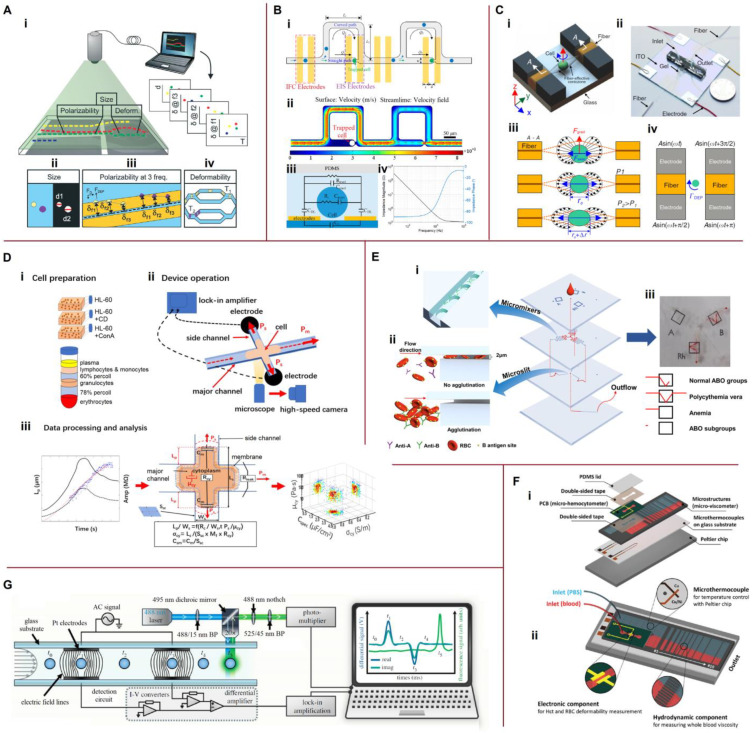
Multifunctional devices. (**A**) Multiparameter intrinsic cytometry: (**i**) Combining different microfluidic modules on one substrate. (**ii**) Size module. (**iii**) Multifrequency nDEP spring module. (**iv**) Deformability module. Reproduced with permission from [177]. Copyright 2018, The Royal Society of Chemistry. (**B**) Combination of impedance flow cytometry (IFC) and electric impedance spectroscopy (EIS): (**i**) Device design. (**ii**) Flow rate distribution in the channels. (**iii**) Electrical model of a single cell. (**iv**) Impedance magnitude and phase spectrum vs. frequency. Reproduced with permission from [178]. Copyright 2019, American Chemical Society. (**C**) Optical stretching and electrorotation of a single cell: (**i**) design and (**ii**) photograph of the chip. (**iii**) Working principle of optical stretch and (**iv**) electrorotation. Reproduced with permission from [179]. Copyright 2020, the author(s). (**D**) Characterization of single-cell cytoplasmic viscosity, cytoplasmic conductivity, and specific membrane capacitance: (**i**) cell preparation, (**ii**) device operation, and (**iii**) data processing and analysis. Reproduced with permission from [180]. Copyright 2020, Springer-Verlag GmbH Germany, part of Springer. (**E**) Four-layer multifunctional microfluidic device from the top to bottom layer: (**i**) Division of RBCs into three serpentine reaction channels. (**ii**) Agglutinated RBCs blocked by the microslots. (**iii**) Resulting red lines in the observation area. Reproduced with permission form [181]. Copyright 2020 American Chemical Society. (**F**) Schematic of physiometer for measuring whole blood viscosity, hematocrit, and RBC deformability: (**i**) physiometer assembly and (**ii**) components. Reproduced with permission from [171]. Copyright 2019, The Royal Society of Chemistry. (**G**) Identification of malaria-parasite-infected cells using combined impedance and fluorescence data. Reproduced with permission from [182]. Copyright 2018, the authors.

Examples of multifunctional microfluidic devices are shown in Figure 10.

Apichitsopa et al. [177] demonstrated a proof-of-concept implementation that could measure five intrinsic single-cell markers, including size, deformability, and polarizability, at three frequencies (Figure 10A). Their cell-tracking cytometry included three primary subsystems: a microfluidic platform, microscope imaging, and image processing (Figure 10A(i)), combining the multifrequency nDEP spring module, the deformability module (in which cells deform as they transit through narrow channels), and a machine-learning algorithm to characterize the cell. The cell sizes (*d*_1_, *d*_2_) in the size module were obtained optically from images as cells flowed through the modules (Figure 10A(ii)). In the multifrequency nDEP spring module, cells driven by hydrodynamic flow forces experienced opposing nDEP forces (Figure 10A(iv)), whose balance was achieved when they approached coplanar electrodes and reached different equilibrium positions (*δ_f1_*, *δ_f2_*, *δ_f3_*) depending on their polarizability at the applied frequency. Consequently, the measured equilibrium positions were considered to be characteristic of the polarizability. The equilibrium positions were measured three times at three different frequencies. In the deformability module, the cells deformed as they transited through narrow channels (Figure 10A(v)). The transit time (*T_1_*, *T_2_*) was considered to be a characteristic of cell deformability.

Zhou et al. [183] integrated the differential impedance measurement and constriction-based cell deformability measurement techniques to characterize the mechanical and electrical properties of cells. As in the work cited previously [168], the transit time through the constriction was used to estimate cell deformability. Feng et al. [178] combined impedance flow cytometry (IFC) and EIS in a microfluidic chip (Figure 10B(i)). The device was designed such that the first incoming cell passed the main IFC channel and was then captured in the trapping site for EIS measurement. The next cell passed through the curved path via the following IFC electrodes before being arrested in the trapping area for subsequent EIS measurement. The time variation in the voltage signals was measured for each cell passing through the IFC electrodes (Figure 10B(iii)), and the impedance spectrum of the trapped cell was obtained using EIS (Figure 10B(iv)). Four parameters—namely, the permittivity and conductivity of the cytoplasm and membrane—were extracted by minimizing the squares between the measured impedance and the model’s estimated value.

The measurement of multiphysical parameters using a microfluidic chip with two opposing optical fibers and four 3D electrodes was presented by Huang et al. [179] (Figure 10C). This chip enabled the determination of five biophysical parameters—namely, the shear modulus, steady-state viscosity, relaxation time from the stretching deformation, area-specific membrane capacitance, and cytoplasm conductivity—from the rotation spectra. When the cell flowing into the fiber effective zone along the main channel was captured by the optical force, the fluid stopped. The cell was then stretched along the optical fiber direction by applying a high-power laser (Figure 10C(iii)). After stretching, the laser power was reduced to allow the cell to return to its original morphology. A camera recorded the time-varying stretching and relaxation. The shear modulus, steady-state viscosity, and relaxation time were calculated using a viscoelastic model. The initial data for the calculations were the strain of the cell as a function of time, laser intensity, and refractive indices of the cell and medium, respectively. After measuring the stretch, AC signals were applied to the four 3D electrodes to rotate the cell in-plane (Figure 10C(iv)). A camera was again used to obtain the rotational spectrum. The cytoplasm’s area-specific membrane capacitance and conductivity were calculated based on Equations (20) and (26).

#### 6.4.2. Microfluidic Hematology on a Single Platform

Wang et al. [184] and Liu et al. [180] developed a microfluidic platform to characterize the cytoplasmic viscosity (*µ_cy_*), cytoplasmic conductivity (*σ_cy_*), and specific membrane capacitance (*C_sm_*) of single cells (Figure 10D). Before invading the lateral narrowed channels, the moving cell was pushed through the main microfluidic constriction channel. Two time-dependent parameters were measured: (1) the cell aspiration length (*L_p_*) obtained from image processing and (2) the impedance at frequencies of 100 kHz and 250 kHz between the ends of the lateral channels. Finite-element simulation was used to determine the relationship between the cytoplasmic viscosity (*μ_cy_*) and other parameters in a dimensionless form *L_p_*/*W_s_* = f(*r*/*W_s_*, *t* × *P_s_*/*μ_cy_*), where f is a nonlinear function, *P_s_* is the aspiration pressure at the two ends of the side constriction channel, *W_s_* is the equivalent side-channel width, *r* is the cell radius, and *t* is the time. Cytoplasmic viscosity (*μ_cy_*) was then used as a fitting parameter for the experimental data (*L_p_*), using the nonlinear function f. Cytoplasmic conductivity (*σ_cy_*) and specific membrane capacitance (*C_sm_*) were obtained from impedance data using a theoretical model.

Only a drop of blood obtained from a finger prick needs to be loaded into the multifunctional microfluidic device, as demonstrated by Lin et al. [181] for blood typing and primary screening of blood diseases (Figure 10E). In this device, the microfluidic channels were preloaded with the appropriate antibodies. Pressurized using a hand-operated pump, a drop of blood was pushed through a four-layer microfluidic chip from top to bottom (Figure 10E(i)). However, if the preloaded antibodies (anti-A, anti-B, and anti-D) interacted with injected whole blood, an agglutination reaction that blocked the microslot in the microfluidic channel was triggered (Figure 10E(ii)). The blocked RBCs formed a visible red line that could be easily read to determine the blood type. If the agglutination reaction did not occur in the microchannel, the erythrocytes passed through the microslot. As a result, there was no observable color (Figure 10E(iii)).

Kim et al. [171] introduced a microfluidic-based physiometer to measure whole blood viscosity, hematocrit, and RBC deformability (Figure 10F). The physiometer consisted of a hydrodynamic component (microviscometer) for measuring whole blood viscosity, an electronic component (microhemocytometer) for measuring hematocrit and RBC deformability, and a temperature control system that included microthermocouples and a Peltier chip. Once the whole blood sample was infused, its impedance spectrum was obtained using an electronic component. In the hydrodynamic module, the whole blood viscosity was derived from the input flow rates of blood, and phosphate-buffered saline (PBS) was used as a reference fluid and the channels were filled with blood and PBS. Ten sets of whole blood viscosity readings were successfully obtained for a given flow rate over a wide range of shear rates. The temperature regime was controlled by microthermocouples and maintained using a Peltier chip. Hematocrit was assessed by analyzing the impedance spectrum, and erythrocyte DI was determined from the change in RBC membrane capacitance.

Honrado et al. [183] combined microfluidic impedance cytometry and fluorescence microscopy to characterize changes in the properties of infected RBCs during the malaria infection cycle (Figure 10G). The infected RBCs were identified using a normalized impedance scatter plot and normalized fluorescence distribution. For healthy RBCs, the cytoplasm permittivity, conductivity, and membrane permittivity were determined using a multishell model based on experimental impedance data. The permittivity and conductivity of the parasite cytoplasm and the permittivity of the parasite membrane were also evaluated for infected RBCs. Analysis showed that the membrane capacitance and cytoplasmic conductivity of infected RBCs increased over the course of infection.

A microfluidic device combining a single AC excitation source for combined trapping using nDEP and AC electroporation was proposed by Punjiya et al. [65] The combination of these two phenomena increases the probability that exogenous materials will approach and enter cells.

Sharifi Noghabi et al. [185] reported a microfluidic single-cell analysis to study multidrug resistance inhibition in leukemic cells. A single cell was trapped by a dielectrophoretic force in the retention structure of the chip, remaining in the same position for approximately one hour. The dye accumulation in the multidrug-resistant leukemic cell efflux inhibition was investigated using fluorescence detection systems.

#### 6.4.3. Neural Networks

Recently, there has been much interest in applying neural networks, machine-learning systems, and artificial intelligence in microfluidics to characterize blood cells [121,186,187]. Using two coplanar pairs of liquid electrodes and two pairs of facing electrodes designed around a recurrent neural network, Honrado et al. [188] characterized RBCs and yeasts with a unitary prediction time of 0.4 ms. The network was trained to predict (with reasonable accuracy) cell size, velocity, and cross-sectional position (i.e., lateral and vertical).

Herbig et al. [189] developed a microfluidic device for active label-free sorting of cells based on their bright-field images supported by innovative real-time image processing and deep neural networks. In the proposed device, the cell suspension was pre-focused in a narrow area just 100 μm wide before being focused on the sheath flow in the direction of the narrowing channel, with cells being captured in a deformed state in the backmost part of this channel. The time required for image processing was optimized to analyze up to 300 cells/s. Downstream of the measurement region, the channel bifurcates toward the default and target outputs; the sorting function is performed using SSAWs that push the target cells into the target outlet.

Rizzuto et al. [190] combined microfluidics with machine-learning algorithms to classify erythrocytes in rare hereditary hemolytic anemia (RHHA). In RHHA, the spleen plays a key role, being the organ responsible for the premature removal of defective erythrocytes. Hence, a microfluidic device mimicking the slits of the spleen red pulp area and video data analysis were combined to characterize erythrocytes.

Jou et al. [191] developed an automatic platform based on a nanostructured microfluidic chip for the isolation and identification of CTCs. The cell analysis tools (CAT) system was designed to identify target cells according to the immunofluorescence staining of cells using pre-set parameters and a deep-learning artificial intelligence function.

Baur et al. [192] proposed magnetic flow cytometers wherein cells were labelled with magnetic nanoparticles to assign a magnetic fingerprint to each cell. They used a magnetoresistive half-bridge sensor integrated into a direct microfluidic channel for cell signal detection and a convolutional neural network to analyze single-cell as well as multicell events. The developed framework made the determination of cell concentrations and hydrodynamic cell diameters possible.

It can be concluded that interdisciplinary methods and multiparameter devices make it possible to determine various physical properties of blood and blood cells and can make a significant contribution to microfluidic hematology laboratories. However, the introduction of such methods and devices into everyday medical practice requires great effort.

## 7. Conclusions and Perspectives

The microfluidic characteristics of blood and blood cells are determined to promote the health and life of patients. Currently, microfluidic devices can reproduce many routine laboratory blood tests. Publications on this topic range from fundamental research to clinical applications.

The further development of microfluidic systems to characterize blood and blood cells has evolved in two directions. The first direction has been the design of portable devices suitable for POC diagnostics, real-time personalized blood analysis, and/or rapid clinical tests. Such devices must be easy to use and preferably perform multiple tests. The second direction has been the integration of various microfluidic platforms into a hematology laboratory on a chip, integrating biological, chemical, medical, and physical research. Such integrated systems must be equipped with precision measuring instruments, external optical systems, and powerful software.

In this review, we focused on the contributions of physics to the microfluidic hematology laboratory, confirming the trend to integrate hydrodynamic, optical, electromagnetic, and/or acoustic methods into a single microfluidic platform. The combination of different methods increases the efficiency and throughput of cell separation, improves the accuracy of microfluidic cytometry, and allows the determination of various physical properties and biological characteristics of blood and blood cells. Our review has demonstrated that most physical parameters can only be measured by combining different fields and influences. The separation of blood cells provides a qualitative comparison of the physical properties of different cells (e.g., harder or softer, larger or smaller, nDEP or pDEP).

We noted the differences between the physical properties and biophysical characteristics of blood. Biophysical characteristics depend on both the physical properties of the test material and the parameters of the measuring device, while physical properties do not depend on the experimental conditions. Examples of biophysical characteristics include the blood impedance for a given electrode configuration and the deformability of RBCs in a constriction-based microfluidic sensor. Relevant examples of physical parameters include conductivity (or complex effective permittivity) and spring constant (or Young’s modulus). We believe that physical properties are the most reliable characteristics of blood and blood cells. Particular attention was paid to the physical quantities that are actually measured by the proposed devices (e.g., transit time, light intensity, and velocity profile) and the parameters that are determined as a result of experiments (deformation index, aggregation index, viscosity, etc.).

Microfluidic devices measure nearly all the properties listed in Figure 1. Exceptions are surface tension, osmotic pressure, and thermodynamic properties. Modern micromechanical technologies make it possible to set and control the temperature; however, the temperature dependence of various parameters has not yet been sufficiently studied.

Looking ahead, there is a growing trend toward using neural networks, machine-learning systems, artificial intelligence, and edge computing to characterize blood cells. We believe that progress in the study of the physical properties of blood will be associated with the use of new hemocompatible materials [193], hydrogels [194,195], and 4D printing technology [196,197]. Self-driven micro-/nanorobots may be involved in cell and particle manipulations [198,199]. Unfortunately, most of the physical properties and biophysical characteristics of blood and blood cells are not included in the list of general blood tests. Currently, there is an accumulation of data on the correlations between the physical properties of blood cells and various diseases. We believe that microfluidic measurements of physical properties will become routine blood tests in the future.

## Data Availability

Not applicable.

## Abbreviations

AI	Aggregation index
CTC	Circulating tumor cell
CM factor	Clausius–Mossotti factor
DEP	Dielectrophoresis
DI	Deformation index
DLD	Deterministic lateral displacement
dRT-DC	Dynamic real-time deformability cytometry
ESR	Erythrocyte sedimentation rate
HCT	Hematocrit
IFC	Impedance flow cytometry
iRBC	Infected red blood cell
MCH	Mean corpuscular hemoglobin
MCHC	Mean corpuscular hemoglobin concentration
MCV	Mean corpuscular volume
MNP	Magnetic nanoparticle
μ-LaFF	Microfluidic lateral flow filtration
μ-PIV	Microparticle image velocimetry
nDEP	Negative dielectrophoresis
NIQPM	Non-interferometric quantitative phase microscopy
PA	Photoacoustic
PAFC	Photoacoustic flow cytometry
PBMC	Peripheral blood mononuclear cell
pDEP	Positive dielectrophoresis
PBS	Phosphate-buffered saline
PIV	Particle image velocity
PLT	Platelet
POC	Point of care
R-TPM	Rolling tomographic phase microscopy
RBC	Red blood cell
RT-DC	Real-time deformability cytometry
SAWs	Surface acoustic waves
SSAWs	Standing surface acoustic waves
TPM	Tomographic phase microscopy
TSAWs	Traveling pulsed surface acoustic waves
WBC	White blood cell
